# Functionally Redundant RXLR Effectors from *Phytophthora infestans* Act at Different Steps to Suppress Early flg22-Triggered Immunity

**DOI:** 10.1371/journal.ppat.1004057

**Published:** 2014-04-24

**Authors:** Xiangzi Zheng, Hazel McLellan, Malou Fraiture, Xiaoyu Liu, Petra C. Boevink, Eleanor M. Gilroy, Ying Chen, Kabindra Kandel, Guido Sessa, Paul R. J. Birch, Frédéric Brunner

**Affiliations:** 1 Department of Biochemistry, Centre for Plant Molecular Biology, Eberhard Karls University, Tübingen, Germany; 2 Division of Plant Sciences, University of Dundee (at James Hutton Institute), Invergowrie, Dundee, United Kingdom; 3 Cell and Molecular Sciences, The James Hutton Institute, University of Dundee, Invergowrie, Dundee, United Kingdom; 4 Department of Molecular Biology and Ecology of Plants, Tel Aviv University, Tel Aviv, Israel; Oregon State University, United States of America

## Abstract

Genome sequences of several economically important phytopathogenic oomycetes have revealed the presence of large families of so-called RXLR effectors. Functional screens have identified RXLR effector repertoires that either compromise or induce plant defense responses. However, limited information is available about the molecular mechanisms underlying the modes of action of these effectors *in planta*. The perception of highly conserved pathogen- or microbe-associated molecular patterns (PAMPs/MAMPs), such as flg22, triggers converging signaling pathways recruiting MAP kinase cascades and inducing transcriptional re-programming, yielding a generic anti-microbial response. We used a highly synchronizable, pathogen-free protoplast-based assay to identify a set of RXLR effectors from *Phytophthora infestans* (PiRXLRs), the causal agent of potato and tomato light blight that manipulate early stages of flg22-triggered signaling. Of thirty-three tested PiRXLR effector candidates, eight, called Suppressor of early Flg22-induced Immune response (SFI), significantly suppressed flg22-dependent activation of a reporter gene under control of a typical MAMP-inducible promoter (*pFRK1-Luc*) in tomato protoplasts. We extended our analysis to *Arabidopsis thaliana*, a non-host plant species of *P. infestans*. From the aforementioned eight SFI effectors, three appeared to share similar functions in both Arabidopsis and tomato by suppressing transcriptional activation of flg22-induced marker genes downstream of post-translational MAP kinase activation. A further three effectors interfere with MAMP signaling at, or upstream of, the MAP kinase cascade in tomato, but not in Arabidopsis. Transient expression of the SFI effectors in *Nicotiana benthamiana* enhances susceptibility to *P. infestans* and, for the most potent effector, SFI1, nuclear localization is required for both suppression of MAMP signaling and virulence function. The present study provides a framework to decipher the molecular mechanisms underlying the manipulation of host MAMP-triggered immunity (MTI) by *P. infestans* and to understand the basis of host versus non-host resistance in plants towards *P. infestans*.

## Author Summary

*Phytophthora* species are among the most devastating crop pathogens worldwide. *P. infestans* is a pathogen of tomato and potato plants. The genome of *P. infestans* has been sequenced, revealing the presence of a large number of host-targeting RXLR effector proteins that are thought to manipulate cellular activities to the benefit of the pathogen. One step toward disease management comprises understanding the molecular basis of host susceptibility. In this paper, we used a protoplast-based system to analyze a subset of *P. infestans* RXLR (PiRXLR) effectors that interfere with plant immunity initiated by the recognition of microbial patterns (MAMP-triggered immunity - MTI). We identified PiRXLR effectors that suppress different stages early in the signaling cascade leading to MTI in tomato. By conducting a comparative functional analysis, we found that some of these effectors attenuate early MTI signaling in Arabidopsis, a plant that is not colonized by *P. infestans*. The PiRXLR effectors localize to different sub-cellular compartments, consistent with their ability to suppress different steps of the MTI signaling pathway. We conclude that the effector complement of *P. infestans* contains functional redundancy in the context of suppressing early signal transduction and gene activation associated with plant immunity.

## Introduction

Plants possess innate defense mechanisms to resist microbial infection [1], [2]. Efficient plant disease resistance is based on two evolutionarily linked layers of innate immunity. One layer involves cell surface transmembrane receptors that recognize invariant microbial structures termed pathogen- or microbe-associated molecular patterns (PAMPs/MAMPs), hereafter referred to as MAMPs [3]–[5]. MAMPs are not only shared by particular pathogen races, but are broad signatures of a given class of microorganisms. They constitute evolutionarily conserved structures that are unique to microorganisms and have important roles in microbial physiology. Typical MAMPs include lipopolysaccharides (LPS) of Gram-negative bacteria, bacterial flagellin and fungal cell wall-derived carbohydrates or proteins, some of which were shown to trigger plant defense in a non-cultivar-specific manner [3], [6]. The best-studied MAMP receptor in plants is FLAGELLIN-SENSITIVE 2 (FLS2) from Arabidopsis, a receptor-like kinase (RLK) with extracellular leucine-rich repeat domains [7]. The 22 amino acid peptide (flg22) corresponding to the highly conserved amino-terminus of flagellin is sufficient to trigger immune responses in Arabidopsis, tomato, tobacco and barley but not in rice [8]–[12]. Although different MAMPs are perceived by different receptors, convergent early-signaling events, including MAP kinase activation and specific defense-gene induction, have been observed in Arabidopsis plants and protoplasts [13]–[15].

Suppression of flg22-induced defenses by bacterial virulence effectors suggests that manipulation of MAMP-triggered immunity (MTI) in plants is a key strategy for successful pathogens to grow and multiply (reviewed in [16]–[19]). A major target of bacterial effectors is the plant MAP kinase cascade, probably because of the central role of MAP kinase signaling in MTI. The *Pseudomonas syringae* effector HopAI1 displays phosphothreonine lyase activity and inactivates MPK3, MPK6, and MPK4 in Arabidopsis by dephosphorylating them [20]. *P. syringae* effector HopF2 blocks MAMP-induced signaling by targeting MKK5, a MAP kinase activating MPK3/MPK6, through a different mechanism of action i.e. ADP-ribosylation [21]. Bacterial effectors can also suppress MAP kinase signaling by targeting the pattern recognition receptor complex as illustrated by the *P. syringae* effectors AvrPto and AvrPtoB that block FLS2-mediated signal transduction in *Arabidopsis* and tomato [22]–[24]. Other effectors appear to act downstream of the activation of the MAPK cascade by blocking the expression of defense-associated genes in the nucleus. Such an effector is XopD from *Xanthomonas campestris* that inhibits the activity of the transcription factor MYB30, resulting in suppression of basal immune responses and promotion of pathogen growth [25], [26].

Unlike bacterial effectors, little is known about the molecular functions of effectors from eukaryotic plant pathogens. It remains to be demonstrated whether these pathogens have evolved effectors that subvert early-induced MTI signaling above, at, or immediately downstream of MAP kinase cascades. Oomycetes, including downy mildews and *Phytophthora* species, establish intimate association with host plant cells through structures such as appressoria, infection vesicles and haustoria, which are believed to facilitate the delivery of effectors into the host cytoplasm [27]. The genome sequences of *Phytophthora sojae*, *P. ramorum*, *P. infestans* and *Hyaloperonospora arabidopsidis* are published [28]–[30]. Each genome encodes several hundred putative RXLR effectors. Most oomycete Avirulence (Avr) proteins characterized so far carry a signal peptide followed by a conserved motif centered on the consensus RXLR-(EER) sequence, where X is any amino acid [31]. It has been shown that the RXLR peptide motif acts as a host-targeting signal for translocation into plant cells [31], [32].

Amongst the best-characterized oomycete RXLR effectors are AVR3a, AVRblb2 and PITG_03192 from *P. infestans*, AVR1b and AVR3b from the soybean pathogen *P. sojae* and ATR1 and ATR13 from *H.* arabidopsidis [33]–[47]. *P. infestans Avr3a* alleles encode secreted proteins of 147 amino acids that differ in two residues which determine recognition; only the isoform AVR3a^KI^ is recognized by the potato resistance protein R3a, whereas AVR3a^EM^ evades detection by R3a. When expressed in *Nicotiana benthamiana* cells, AVR3a suppresses host cell death induced by the elicitin INF1, a typical MAMP [35], [37]. It has since been shown to suppress cell death elicited by perception of a range of pathogen molecules by direct interaction with, and stabilization of, the plant E3 ligase CMPG1 [36], [42]. The *Avrblb2* gene family is highly polymorphic and different forms/alleles are present in different *P. infestans* isolates. Sequence alignment of the deduced amino acid sequences of the *Avrblb2* family members showed that the C-terminal effector domain undergoes positive selection, which is strong evidence for co-evolution with host resistance and/or target proteins [44]. The amino acid residue at position 69 was shown to be crucial for recognition by the cognate resistance protein Rpi-blb2 [44]. AVRblb2 was shown to block the secretion of a C14 cysteine protease that is involved in plant resistance against *P. infestans* [38]. Recently, the RXLR effector PITG_03192 has been shown to enhance *P. infestans* colonization of *N. benthamiana* by its interaction with NAC DNA binding proteins at the host endoplasmic reticulum, preventing their re-localization into the nucleus following pathogen perception [43]. Suppression of MTI has also been reported for ATR1 and ATR13 in Arabidopsis [47]. Nevertheless, for the majority of RXLR effectors, their biological functions and potential host targets are unknown.

Transient expression in protoplasts has proven fast and reliable for studying the function of bacterial type III effectors that suppress early MAMP signaling [48], [49]. Moreover, the assay allows the measurement of synchronized responses and it does not require the use of bacteria for protein or DNA transfer into the host cell. In addition, the protoplast system offers the possibility to test large sets of effectors in a medium-high throughput manner. In this study, we have used tomato mesophyll protoplasts to screen a library of 33 *P. infestans* RXLR effector candidates (PiRXLRs) for their ability to suppress flg22-triggered defense signaling. Our additional aim was to test whether PiRXLRs that suppress early MTI signaling in the host plant tomato retain that ability in the distantly-related non-host plant Arabidopsis. For the experimental read-out we measured the abilities of these effectors to suppress: i) flg22-induced *promoterFLG22-INDUCED RECEPTOR-LIKE KINASE 1 - LUCIFERASE* (*pFRK1-Luc*) reporter gene activity; ii) flg22-induced post-translational MAP kinase activation; and iii) flg22-induced gene expression. In addition, we performed sub-cellular localization studies of fluorescent protein-tagged PiRXLR effectors by confocal microscopy. Finally, we tested the potential of the PiRXLR effectors suppressing early MTI signaling to enhance *N. benthamiana* susceptibility to *P. infestans*. Unraveling the mode-of-action of PiRXLR effectors within plant cells will help to gain insight into the specific mechanisms that coordinate different signaling and metabolic pathways to ensure proper plant development and response to environmental changes or stresses.

## Results

### A subset of RXLR effectors from *P. infestans* suppresses flg22-inducible reporter gene activation in both tomato and Arabidopsis

A prerequisite to performing a screen that would allow us to identify PiRXLR effector candidates suppressing early events of MAMP signaling pathways in both a host (tomato) and a non-host (Arabidopsis) of *P. infestans* was to develop comparative bio-assays. Several components of the flg22-triggered signaling pathway are conserved in Arabidopsis and tomato. SlFLS2, the ortholog of AtFLS2, binds flg22 [50]. The MAP kinase orthologs of AtMPK3 and 6 in tomato are SlMPK3 and 1, respectively [51].

We adapted most of the techniques and materials that were generated for the identification and functional characterization of the *P. syringae* type III effector AvrPto, a well-studied suppressor of early MAMP signaling in both Arabidopsis [48], [49] and tomato [52]. Figure S1 shows that we could reproduce the AvrPto-mediated suppression of early MTI signaling observed in Arabidopsis protoplasts [48]. Moreover, we were able to extend this assay to tomato, and the induction of luciferase activity under control of the flg22-responsive promoter of *FRK1* (*pFRK1-Luc*) was strongly impaired in Arabidopsis and tomato protoplasts expressing AvrPto with a C-terminal Green Fluorescent Protein (GFP) fusion (Figure S1A, B). An inactive AvrPto in which the Gly residue in position 2 is replaced by an Ala (AvrPto G2A-GFP), preventing the myristoylation and membrane localization of the effector protein [53], could not suppress *pFRK1-Luc* activation by flg22 (Figure S1A, B). Furthermore, we confirmed that AvrPto-GFP but not the AvrPto G2A-GFP mutant blocks post-translational activation of flg22-responsive MAP kinases in both protoplast systems (Figure S1 C, D).

We searched for PiRXLR effectors interfering with flg22-induced early immune responses in protoplasts of tomato, a host for *P. infestans*. Thirty-three PiRXLR effector genes, most of which were selected on the basis of their up-regulation during the biotrophic phase of infection [28], [32], [44], were cloned without the native secretion signal peptide into pDONR Gateway vectors (Table S1). We sub-cloned these sequences into Gateway destination vectors of the p2GW7 series to allow transient expression with/without an N-terminally fused GFP tag.

**Figure 1:**
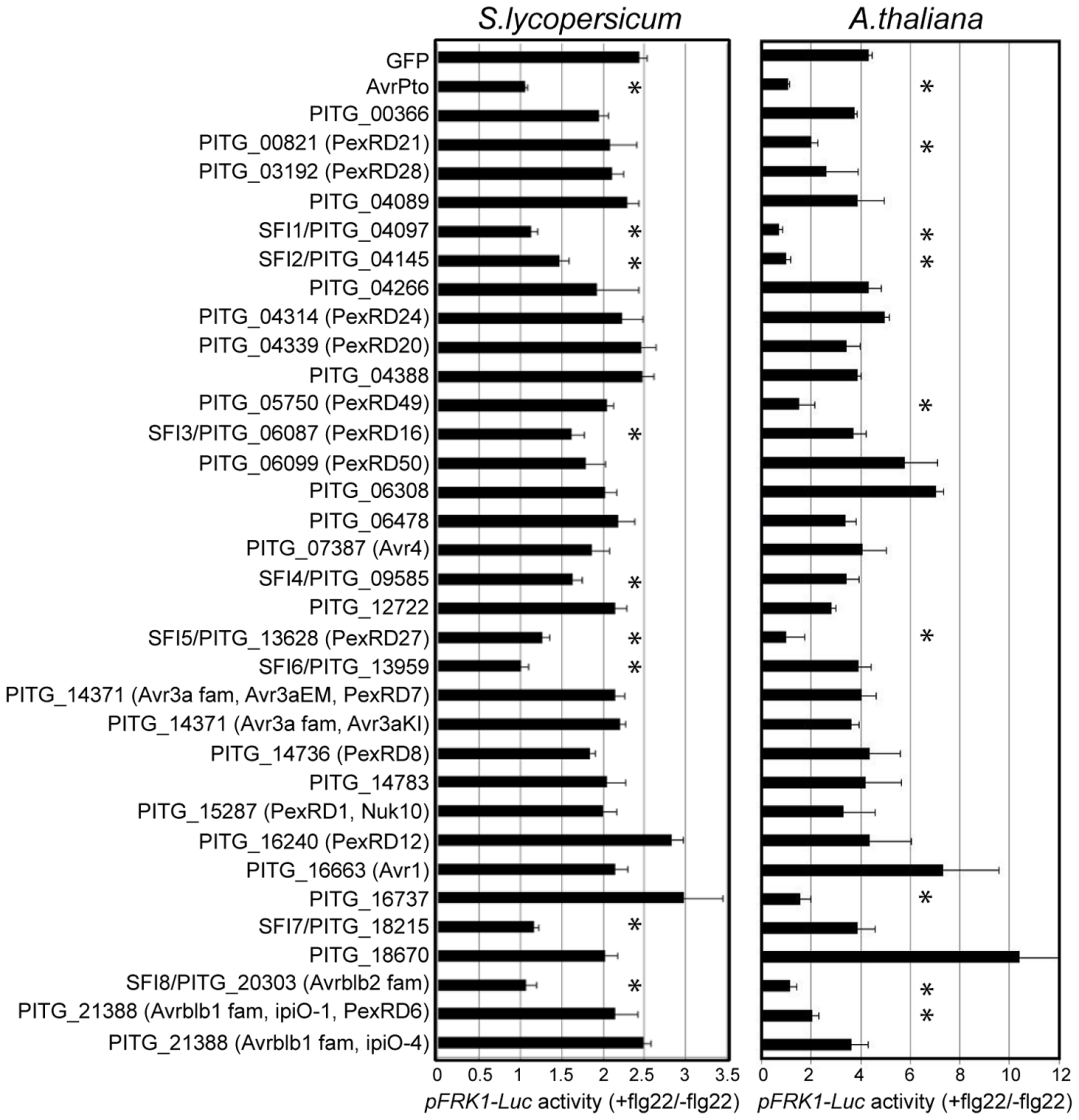
Inhibition of MAMP-inducible reporter gene activation by PiRXLR effectors. Luciferase reporter gene activity in flg22-challenged *S. lycopersicum* and *A. thaliana* protoplasts expressing PiRXLR effector genes. Mesophyll protoplasts were co-transfected with a *p35S-effector* construct (or a *p35S-GFP* control vector) and the two reporter gene constructs *pFRK1-Luc* and *pUBQ10-GUS*. Reporter gene activity was assessed 3 or 6 h later for *S. lycopersicum* and *A. thaliana*, respectively. For each data set, flg22-induced luciferase activity was calculated relative to the untreated sample and was normalized by the corresponding GUS activities in flg22 and untreated sample (*pFRK1-Luc* activity (+flg22/−flg22)). AvrPto was used as a positive control for *pFRK1-Luc* activity suppression. Four independent biological experiments were carried out per effector. Within each experiment three technical replicates were performed. Pooled data are presented as mean ± SEM. Differences in luciferase/GUS activity between control and effector gene-expressing protoplasts were determined using one-way ANOVA followed by Dunnett's multiple comparison test. An asterisk marks data sets with a p-value<0.05.

For the initial read-out, we measured *pFRK1-Luc* activity upon flg22 treatment. Of the 33 PiRXLR effectors screened, 8 (PITG_04097, PITG_04145, PITG_06087, PITG_09585, PITG_13628, PITG_13959, PITG_18215 and PITG_20303) reduced consistently and reproducibly flg22-induced *pFRK1-Luc* activation in tomato protoplasts, when compared to control protoplasts expressing only GFP (p-value<0.05 - Figure 1: *S.lycopersicum*). We named these effectors Suppressor of early Flg22-induced Immune response (SFI) 1 to 8, respectively. Protoplast staining with vital dyes, 24 h after plasmid transformation, showed that the percentage of dead cells is, with the exception of a higher (but non-significant) value for SFI6, similar for each of the tested PiRXLR effectors and the GFP control (Figure S2). Therefore, the suppression of reporter gene activity is not the consequence of a toxic or a programmed cell death process in transformed protoplasts.

Five PiRXLR effectors (SFI1 and SFI5-8) reduced *pFRK1-Luc* activation by flg22 with an efficiency comparable to the bacterial effector AvrPto (+flg22/−flg22≅1). Among PiRXLR effectors with a reported avirulence function in potato, only AVRblb2 (SFI8) [44] was able to suppress flg22-induced *pFRK1-Luc* activity. SFI8 is a representative member of a large family of AVRblb2-related proteins but it bears a Phe residue at position 69 in its sequence and, therefore, is predicted not to be recognized by Rpi-blb2 [44]. Thus, we extended our analysis to three more AVRblb2 family members with either an Ala (PITG_20300 and PITG_04090) or Ile (PITG_04085) at position 69 and crucial for Rpi-blb2-mediated HR (Table S2). Both predicted Rpi-blb2-recognized and -unrecognized isoforms of AVRblb2 equally suppressed reporter gene activation (Figure S3A). Other PiRXLR effectors identified as avirulence proteins such as AVR1 [28], AVR3a [34], AVR4 [54] and AVRblb1/IPI-O1 or IPI-O4 [55], [56] did not interfere with early flg22-induced responses in our assay (Figure 1: *S. lycopersicum*). In the case of AVR3a, both R3a-recognized AVR3a^KI^ and R3a-unrecognized AVR3a^EM^ had no effect on flg22-induced *pFRK1-Luc* activity (Figure 1: *S.lycopersicum*).

Using quantitative real-time PCR (qRT-PCR) we monitored the expression levels of the eight PiRXLR effector genes that suppressed *pFRK1-Luc* activation in tomato protoplasts at different stages of potato infection, relative to their expression in sporangia. Previous expression analyses of *P. infestans* RXLR effector genes showed that, when detected by either qRT-PCR [24] or in microarray experiments [28], [57], they are up-regulated in the first 48–72 hours of infection, i.e. during biotrophy. Transcripts of *SFI1-8* accumulated during the first 48 hours post-inoculation (Figure S4), consistent with a potential role in effector-triggered susceptibility.

We extended our analyses to determine whether PiRXLR effectors that suppress *pFRK1-Luc* activity in the host tomato are able to also suppress such responses in the non-host plant Arabidopsis.

The *pFRK1-Luc* reporter gene assay, which turned out to be more sensitive in Arabidopsis than in tomato, showed that four effectors (SFI1, SFI2, SFI5 and SFI8/AVRblb2) were also able to attenuate activation in Arabidopsis (p-value<0.05 - Figure 1: *A. thaliana*). As observed in tomato, each tested AVRblb2 isoform suppressed reporter gene activation by flg22 in Arabidopsis protoplasts (Figure S3B), whereas AVR3a had no effect (Figure 1: *A. thaliana*). We found a further four effectors (PITG_00821, PITG_05750, PITG_16737 and AVRblb1/PITG_21388) that attenuated the flg22-dependent *pFRK1-Luc* activation only in Arabidopsis (p-value<0.05 - Figure 1: *A. thaliana*). Like in tomato, transient expression of PiRXLR effectors in Arabidopsis protoplasts did not cause significant cell death (Figure S5). One effector, PITG_18670, significantly induced a stronger flg22-dependent *pFRK1-Luc* activity than did the GFP control (p-value<0.05 – Figure 1: *A. thaliana*), but did not do so in the host plant tomato (Figure 1: *S. lycopersicum*). This effector was not pursued further in this work.

**Figure 2:**
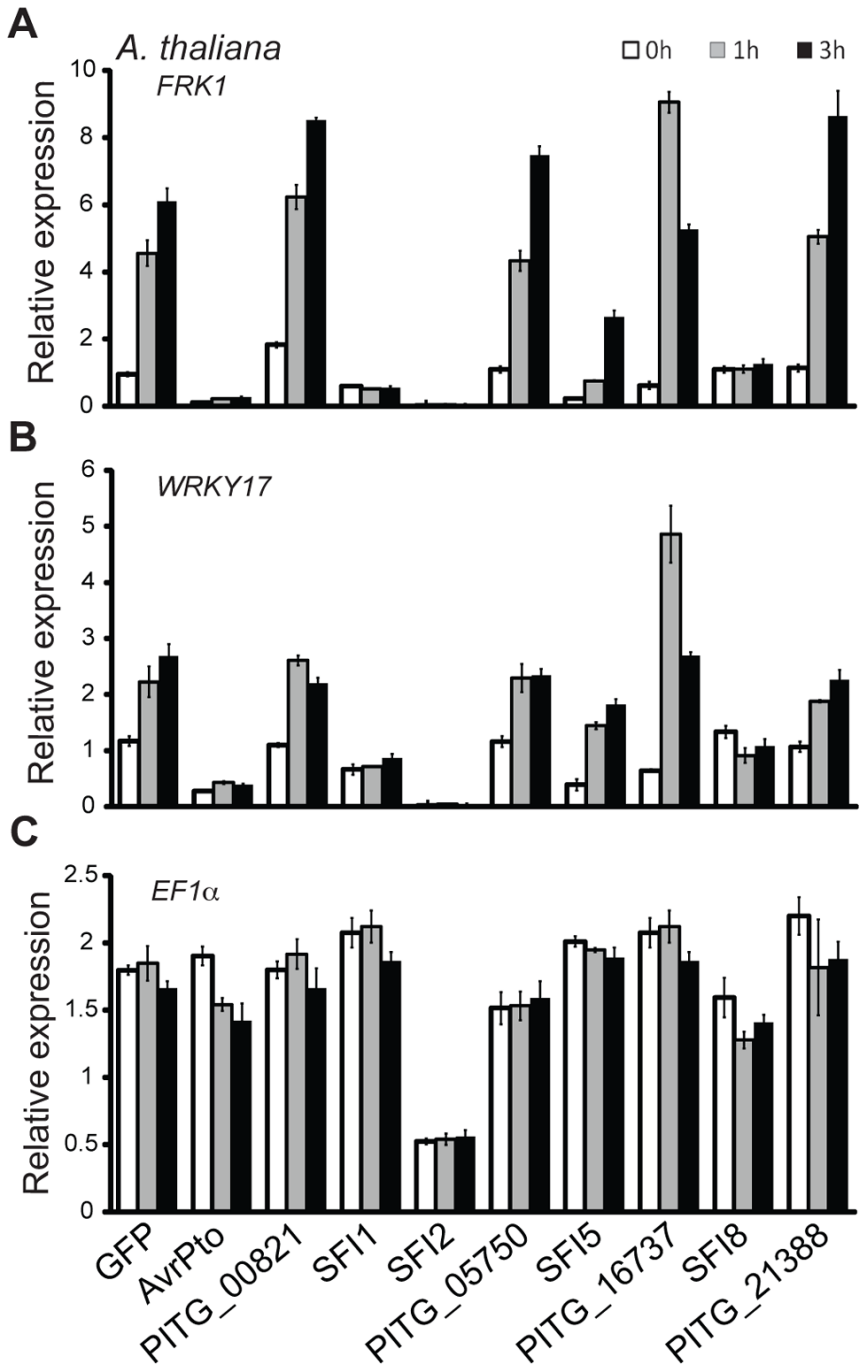
Transcriptional profiling of MAMP-inducible genes in *A. thaliana* protoplasts transfected with SFI effector constructs. (**A–C**) Relative gene expression for the flg22-inducible genes *FRK1* and *WRKY17* (**A, B**) and the housekeeping gene *EF1α* (**C**) was assessed by quantitative real-time polymerase chain reaction (qRT-PCR) 0, 1 and 3 h after protoplasts were exposed to flg22. Transcript levels of the analyzed genes were normalized to the levels of the *Actin* transcript. GFP was used as a negative and AvrPto as a positive control for suppression of gene expression. One representative independent experiment out of four is plotted. Data is presented as mean ± SEM from three technical replicates.

The observation that 4 PiRXLR effectors suppress flg22-mediated *pFRK1-Luc* induction in the non-host plant Arabidopsis, but not in the host plant tomato, was unexpected. This prompted us to test whether all 8 PiRXLR effectors that suppress *pFRK1-Luc* induction in Arabidopsis also inhibit the endogenous expression of early MAMP-regulated genes. First, we measured the level of endogenous *FRK1* in Arabidopsis following flg22 treatment. This experiment confirmed the data obtained in the reporter gene assay with 3 PiRXLR effectors (SFI1, SFI2 and SFI8/AVRblb2) attenuating the up-regulation of *FRK1* expression by flg22 (Figure 2A). In contrast, SFI5, as well as PITG_00821, PITG_05750, PITG_16737 and AVRblb1/PITG_21388, failed to suppress flg22-induced *FRK1* expression (Figure 2A). We extended our analysis to an additional MAMP-induced gene, *WRKY DNA-BINDING PROTEIN 17* (*WRKY17*), and observed that its up-regulation was also notably diminished by SFI1, SFI2 and SFI8/AVRblb2 (Figure 2B), whereas SFI5, PITG_00821, PITG_05750, PITG_16737 and AVRblb1/PITG_21388 again had no effect. The expression of the housekeeping gene *ELONGATION FACTOR 1A* (*EF1α*) was generally not altered. Only with SFI2 did we observe a 2–3 fold decrease of the *EF1α* transcript level, possibly as a consequence of reduced cellular fitness due to effector expression (Figure 2C). Indeed, the expression of all genes tested was barely detectable in the presence of this effector.

Together, our initial results revealed a set of 8 PiRXLR effectors that are candidate suppressors of early flg22-mediated MTI signaling in tomato, and assigned a novel function to the previously described AVRblb2 effector family. Moreover, our data predict that 3 of these PiRXLR effectors target processes contributing to MTI that are conserved in Arabidopsis and tomato. We proceeded to study all 8 effectors that suppress flg22-inducible reporter gene activation in tomato in more detail.

### PiRXLR effectors suppressing flg22-inducible reporter gene activation display similar sub-cellular localizations in tomato and Arabidopsis protoplasts and in *N. benthamiana* leaves

**Figure 3:**
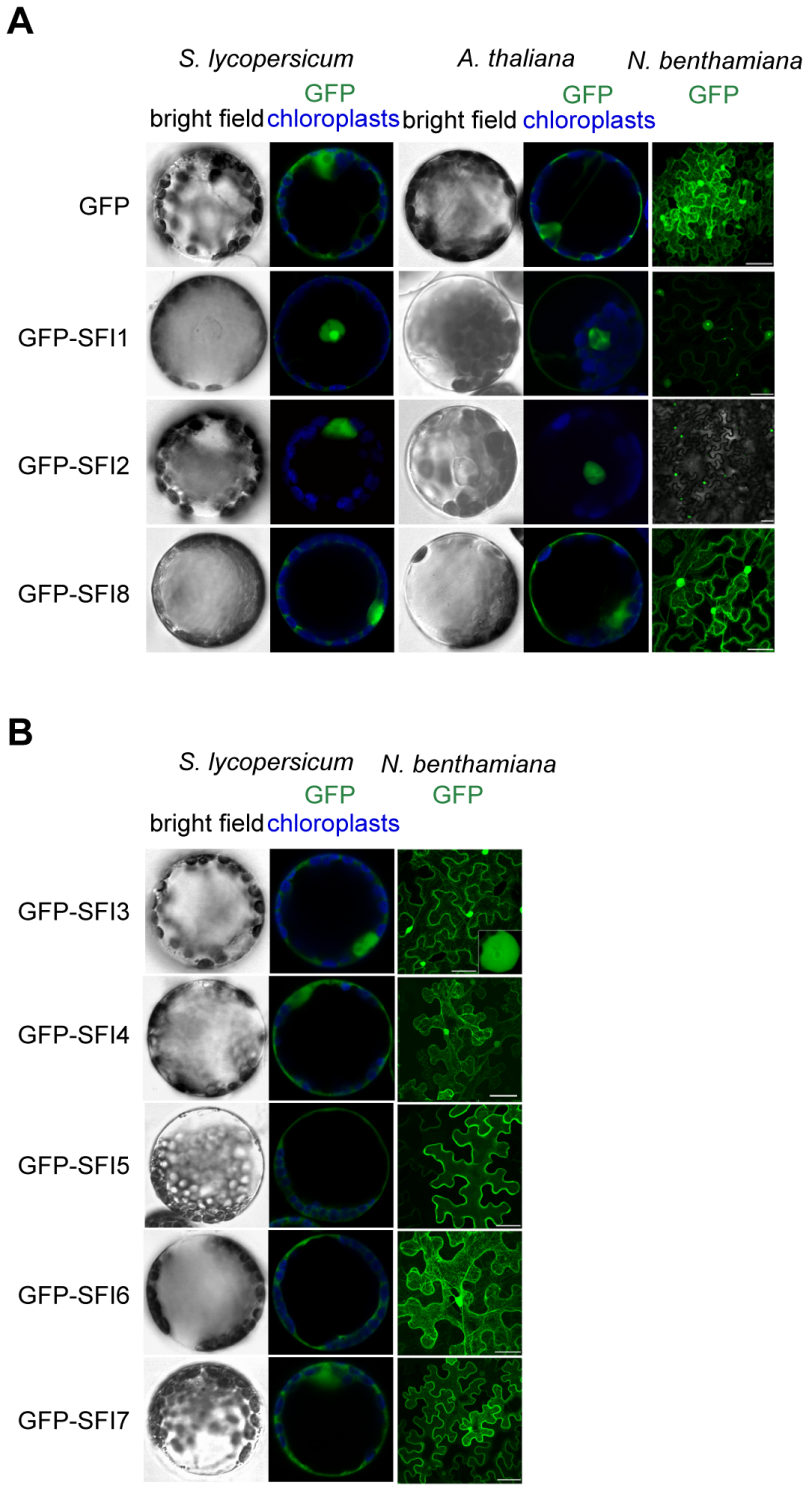
Sub-cellular localization of N-terminally GFP-tagged SFI effectors in *S. lycopersicum* and *A. thaliana* protoplasts and in *N. benthamiana* leaves. (**A, B**) Mesophyll protoplasts and *N. benthamiana* leaves were monitored using confocal microscopy 12 h and 48 h after transfection with a *p35S-GFP-effector* construct, respectively. Representative optical sections of bright field and merged GFP (in green) and chloroplast (in blue) fluorescence images are shown for protoplasts as indicated.

From the initial screen for MTI signaling suppression we hypothesized that the function of 3 PiRXLR effectors (SFI1, SFI2 and SFI8/AVRblb2) may be conserved in both tomato and Arabidopsis while 5 effectors (SFI3, SFI4, SFI5, SFI6 and SFI7) may function specifically in tomato. We expected that the sub-cellular distribution of PiRXLR effectors might provide additional important information about their function in the cell. Therefore, these PiRXLR effectors, N-terminally fused to GFP, were transiently expressed in tomato (all SFI effectors) and Arabidopsis (only SFI1, SFI2 and SFI8/AVRblb2) protoplasts, and in *N. benthamiana* leaves for comparison, and visualized by confocal microscopy (Figure 3). We performed immunoblot analysis to confirm protein expression and stability of intact GFP-fusion proteins (Figure S6), and verified that GFP-tagged PiRXLR effectors were still functional and effectively suppressed *pFRK1-Luc* activity in protoplasts (Figure S7). Most of the GFP-tagged PiRXLR effectors were as active as the un-tagged proteins. Notably, GFP-SFI8/AVRblb2 functioned only weakly or not at all in Arabidopsis, but retained its function in tomato (Figure S7). SFI8/AVRblb2, C-terminally fused to GFP (SFI8-GFP) was also unable to suppress *pFRK1-Luc* activity in Arabidopsis protoplasts (Figure S8).

The sub-cellular localizations of the 3 PiRXLR effectors (SFI1, SFI2 and SFI8/AVRblb2) affecting *pFRK1-Luc/*MAMP gene activation in both tomato and Arabidopsis are similar in each plant species (Figure 3A). GFP-SFI8/AVRblb2 showed nuclear-cytoplasmic localization whereas GFP-SFI1 and GFP-SFI2 localized predominantly in the nucleus, and were also apparent in the nucleolus (Figure 3A). In the case of GFP-SFI1, additional fluorescence signal was observed in the cytoplasm (and possibly at the plasma membrane [PM]) (Figure 3A). The 5 PiRXLR effectors (GFP-SFI3, -SFI4, -SFI5, -SFI6 and -SFI7) with a tomato-specific effect showed different subcellular localizations. GFP-SFI3 was enriched in the nucleus/nucleolus, GFP-SFI4 showed nuclear-cytoplasmic localization, and GFP-SFI5, -SFI6 and -SFI7 showed differing degrees of cytoplasmic localization and association with the PM (Figure 3B), with GFP-SFI5 almost exclusively localized to the PM.

Additional sub-cellular localization studies performed upon Agrobacterium-mediated expression in *N. benthamiana* leaves confirmed the results obtained in protoplasts, suggesting that protoplasts are accurate in reflecting sub-cellular localizations of these effectors *in planta*. Confocal microscopy revealed distinct sub-nuclear localization patterns for the 3 PiRXLR effectors (GFP-SFI1-3) that were predominant in this compartment. GFP-SFI1 appears to localize in the nucleolus, GFP-SFI3 forms a ring around the nucleolus, whereas GFP-SFI2 showed a range of sub-nuclear localizations (Figure S9). The obvious differences in sub-cellular localization between effectors imply that different steps and/or pathways may be targeted by individual effectors that have in common the suppression of flg22-triggered *pFRK1-Luc* activity.

### SFI5, SFI6 and SFI7 suppress flg22-induced post-translational MAP kinase activation in tomato but not in Arabidopsis

We performed an epistatic analysis to find out which step of the flg22-triggered signaling pathway in tomato or Arabidopsis is affected by the PiRXLR effectors that suppressed *pFRK1-Luc*/MAMP responsive gene activation. We conducted immunoblot assays using the p44/42 antibody, raised against phosphorylated MAP kinases, to assess the impact of our effectors on the activation by flg22 of endogenous SlMPK1/3 and AtMPK3/6 in tomato and Arabidopsis protoplasts, respectively. AvrPto was used as a positive control, as it is known to block MTI signaling upstream of the MAP kinase cascade at the FLS2/BAK1 receptor complex [23], [24], [48].

**Figure 4:**
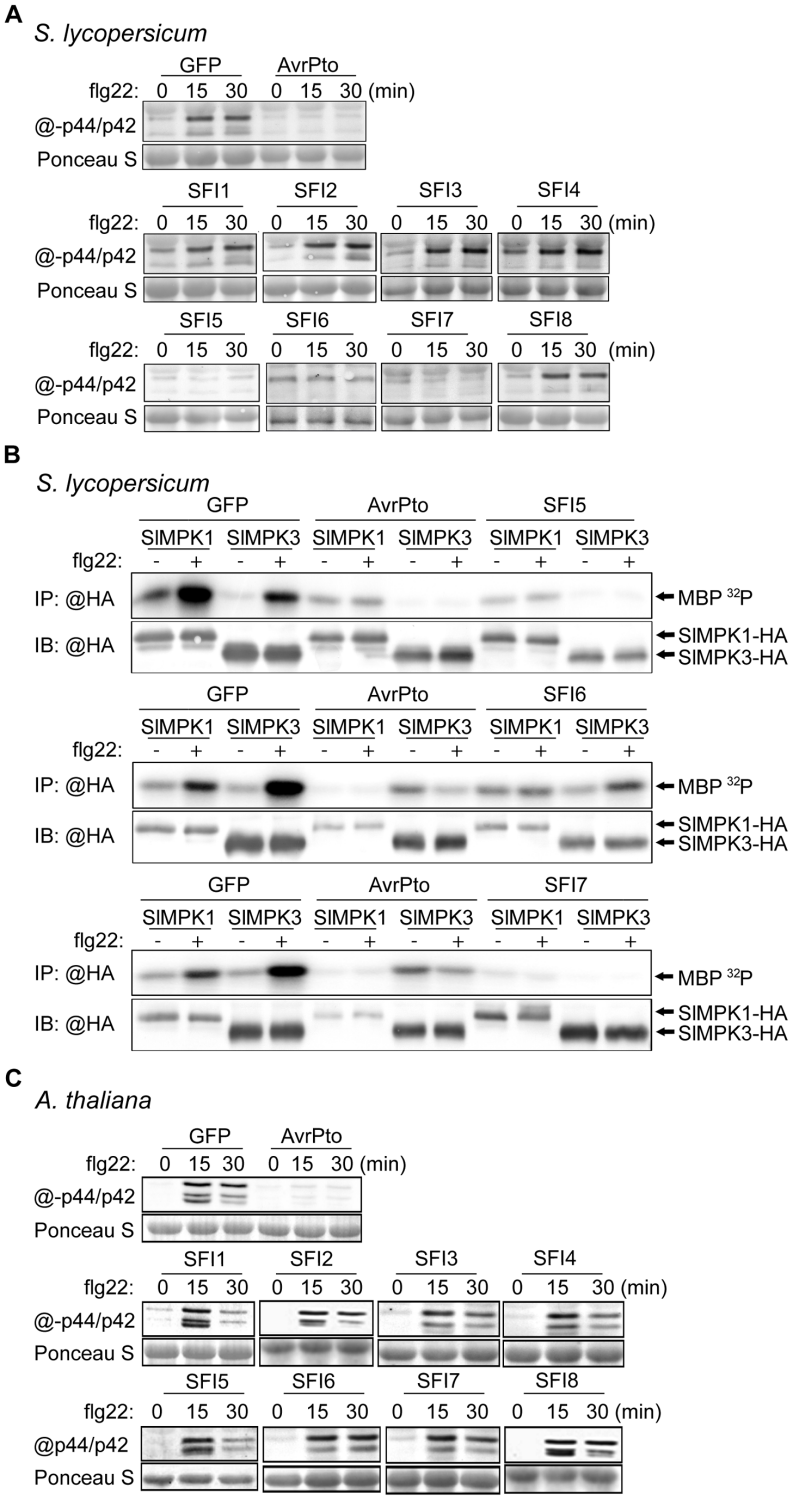
MAP kinase activation upon flg22 treatment in protoplasts expressing SFI effector genes. (**A, C**) Immunoblotting of phosphorylated MAP kinase in *p35S-effector*-transfected *S. lycopersicum* (**A**) and *A. thaliana* (**C**) protoplast samples collected 0, 15 and 30 min after flg22 treatment. Antibody raised against activated MAP kinase p44/p42 was used for detection. The experiments are representative of at least two repeats. Ponceau S staining served as a loading control. (**B**) MAP kinase *in vitro* kinase assay in *S. lycopersicum* protoplasts. GFP, AvrPto, SFI5, SFI6 or SFI7 were co-expressed with hemagglutinin (HA)-tagged tomato MAP kinase SlMPK1 or SlMPK3. HA-tagged MAP kinase were immunoprecipitated with anti-HA antibody for an *in vitro* kinase assay with [γ-^32^P] ATP and myelin basic protein (MBP) as phosphorylation substrate. The lower panel presents an immunoblot with anti-HA antibody showing the expression of HA-tagged proteins. The upper panel shows an autoradiography visualizing MBP phosphorylation (MBP ^32^P) in the presence of immunoprecipitated MAP kinase. The experiment was repeated twice with similar results.

In tomato, 3 effectors (SFI5-SFI7) consistently suppressed flg22-dependent post-translational MAP kinase activation (Figure 4A). We confirmed this result by performing transient expression of HA-tagged SlMPK1 and SlMPK3 in protoplasts followed by immunoprecipitation and *in vitro* MAP kinase assay (Figure 4B). In contrast, none of the 8 SFI effectors attenuated flg22-dependent post-translational MAP kinase activation in Arabidopsis (Figure 4C). This suggests that the effectors (SFI1, SFI2 and SFI8/AVRblb2) that were shown to attenuate flg22-induced gene activation in both tomato and Arabidopsis are most likely doing so downstream of MAP kinase activation. In the case of SFI5, the demonstration that it attenuates MAP kinase activation only in tomato (Figure 4A, 4C) is consistent with the observation that, although this effector suppressed *pFRK1-Luc* activation in Arabidopsis, it failed to suppress flg22-mediated up-regulation of endogenous *FRK1* in that plant.

### SFI5 and SFI6 specifically suppress flg22-induced MAP kinase signaling, whereas SFI7 also partially attenuates INF1-triggered cell death

To further elucidate the molecular mechanism(s) underlying the mode of action of SFI5-SFI7 in suppressing flg22-induced post-translational MAP kinase activation in tomato, we performed gain-of-function experiments using components that activate the MAP kinases SlMPK1 and SlMPK3 in the absence of flg22 signal. The ectopic expression of known key players of MAMP-signaling pathways, such as MAPK kinases and MAPKK kinases [48], [58] have helped to elucidate the steps at which bacterial effectors such as AvrPto interfere with MTI in Arabidopsis [48], [59].

**Figure 5:**
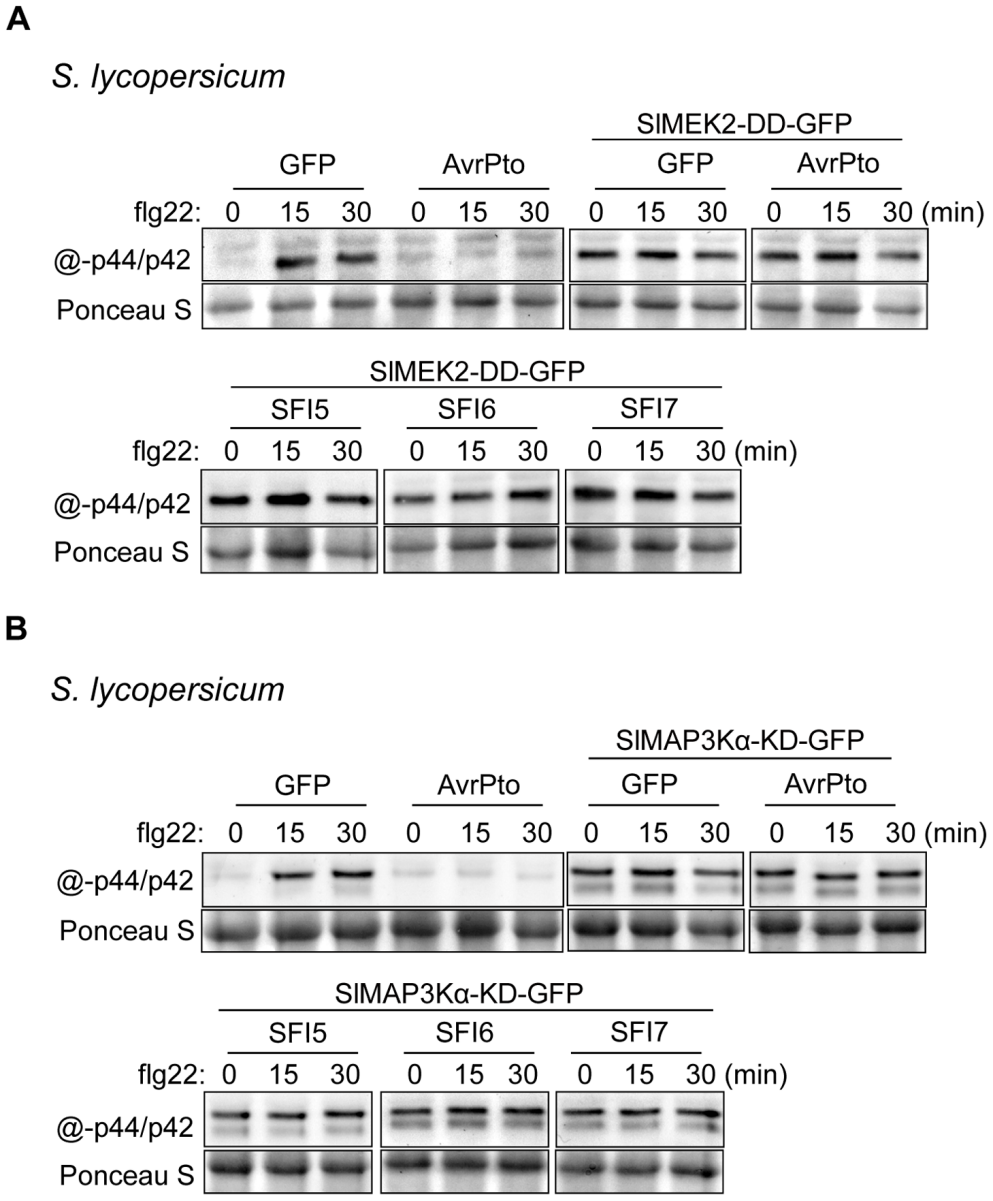
Epistatic analysis of MAP kinase activation upon flg22 treatment in *S. lycopersicum* protoplasts expressing SFI effector genes. Immunoblotting of phosphorylated MAP kinase in *p35S-effector*- and *p35S-SlMEK2-DD-GFP-* (**A**) and in *p35S-effector*- and *p35S-SlMAP3Kα-KD-GFP-* (**B**) co-transfected *S. lycopersicum* protoplast samples collected 0, 15 and 30 min after flg22 treatment. Antibody raised against activated MAP kinase p44/p42 was used for detection. The experiments are representative of at least two repeats. Ponceau S staining served as a loading control.

In tomato and other solanaceous plants, MAP kinase signaling cascades are best studied in the context of programmed cell death (PCD) associated with effector-triggered immunity [51], [60], [61]. In *N. benthamiana*, PCD triggered by perception of the *P. infestans* MAMP INF1 requires NbMKK1 and its interaction with SIPK (salicylic acid-induced protein kinase; an ortholog of SlMPK1) [62]. The role of MAPKK kinases in tomato immunity is only documented for SlMAP3Kα and SlMAP3Kε [60], [61] and the best characterized MAPK kinases are SlMEK1 and SlMEK2 [60]. Whether these kinases contribute to flg22-triggered signaling in tomato is unknown. As shown in Figure S10, transient expression in tomato protoplasts of a constitutively active SlMEK2 (SlMEK2-DD), or the kinase domain of SlMAP3Kα (SlMAP3Kα-KD), led to post-translational activation of SlMPK1 and SlMPK3 in the absence of flg22. The constitutively active SlMEK1 (SlMEK1-DD) and kinase domain of SlMAP3Kε (SlMAP3Kε-KD) did not activate SlMPK1 and SlMPK3. The expression of the constitutively active SlMEK2 (SlMEK2-DD) and the kinase domain of SlMAP3Kα (SlMAP3Kα-KD) overrode the suppression of flg22-dependent activation of SlMPK1 and SlMPK3 by SFI5-SFI7 (Figure 5A, 5B). These results indicate that the three effectors suppress the signaling cascade very early; either upstream of MAPKK kinase activation, or specifically at the MAPK- and/or MAPKK kinase(s) involved in flg22 signaling. This is consistent with association of these effectors with the plant plasma membrane, where they may interfere with the earliest components of MAMP perception or signal transduction.

**Figure 6:**
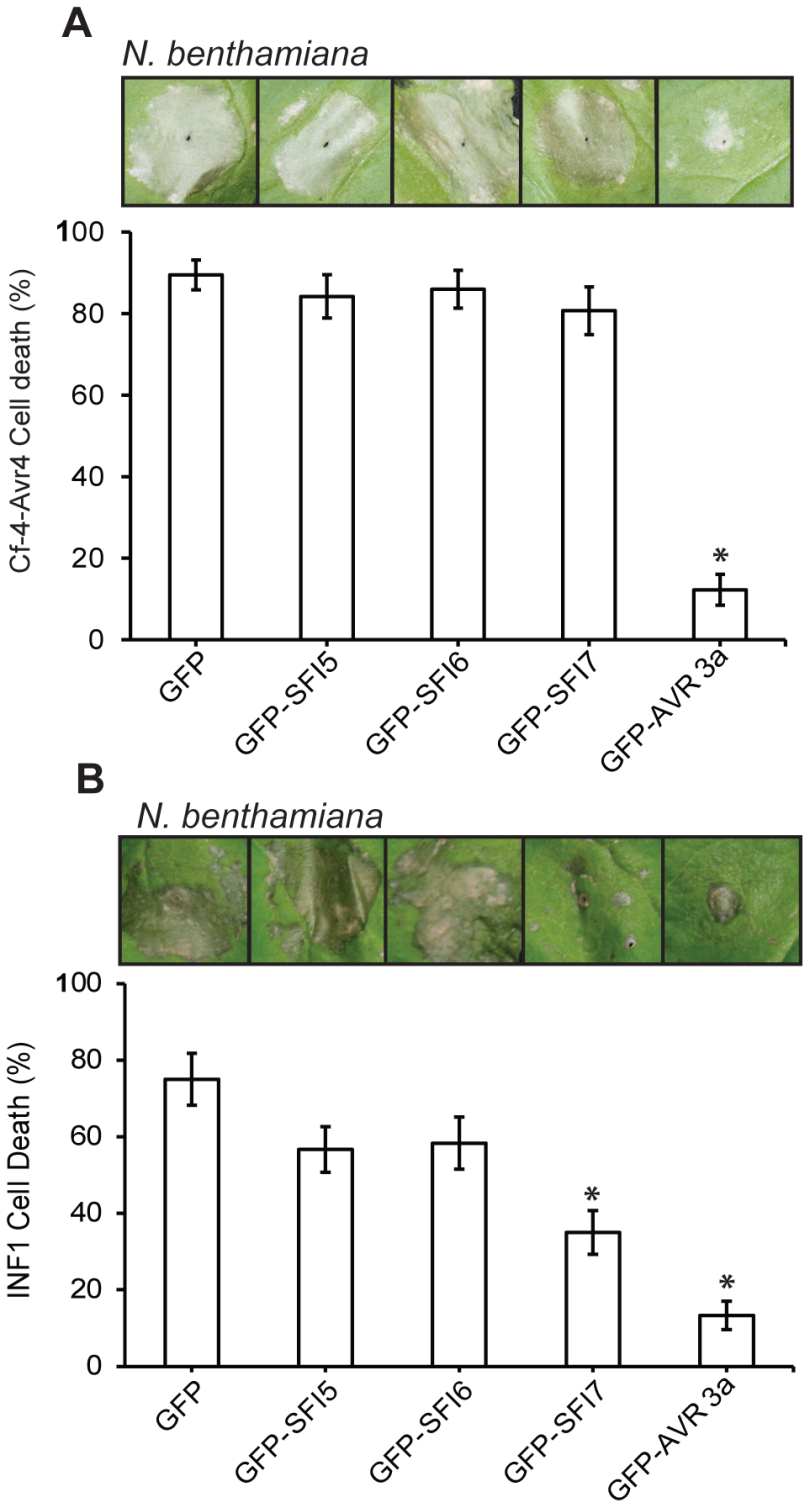
Effect of GFP-SFI5, GFP-SFI6 and GFP-SFI7 on PCD triggered by INF1 or by co-expression of Cf-4 with *Cladosporium fulvum* Avr4. (**A**) Percentage of inoculation sites showing confluent cell death at 7 days post-infiltration of *Agrobacterium* strains expressing each GFP-effector fusion protein with a strain expressing Cf-4 and Avr4. (**B**) Percentage of inoculation sites showing confluent cell death at 7 days post-infiltration of *Agrobacterium* strains expressing each GFP-effector fusion protein with a strain expressing INF1. Results in (**A**) and (**B**) represent five biological replicates, each involving 18 inoculation sites. Error bars represent SEM. * represents statistical significance (p<0.01) using one-way ANOVA.

Since in *N. benthamiana* PCD triggered by perception of the MAMP INF1 [62], or perception of *Cladosporium fulvum* effectors Avr4/9 by tomato Cf-4/9 receptors [60], [61], involves MAP kinase cascades, we tested whether SFI5-SFI7 were able to suppress either PCD event. In contrast to AVR3a, which is known to suppress PCD triggered by INF1 or by co-expression of Cf-4/Avr4 ([42]); Figure 6a, 6b – p-value<0.01), GFP-SFI5 and GFP-SFI6 did not attenuate PCD triggered by either recognition event (Figure 6A, 6B). However, whereas GFP-SFI7 also failed to suppress Cf-4/Avr4-mediated PCD (Figure 6B), this effector significantly attenuated INF1-mediated PCD, albeit less efficiently than AVR3a (Figure 6B – p-value<0.01). Our results indicate that SFI5 and SFI6 display functional specificity by targeting the flg22/FLS2 MAP kinase cascade, but not suppressing MAP kinase cascades leading to Cf-4- or INF1-mediated PCD, whereas SFI7 has a broader suppressive effect which includes INF1- but not Cf-4-mediated PCD.

### PiRXLR effectors suppressing early MTI signaling contribute to *P. infestans* virulence

**Figure 7:**
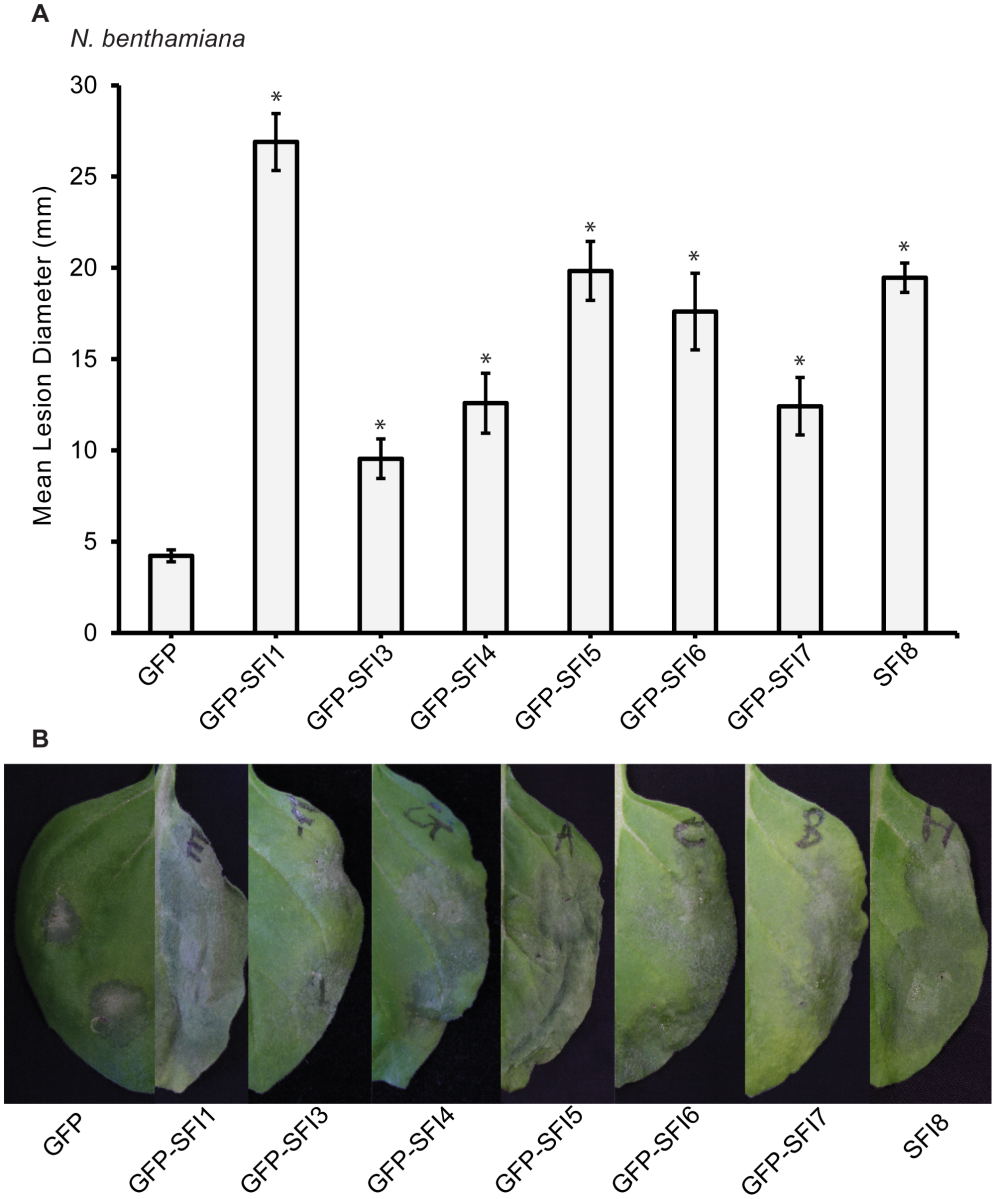
Effect of transient expression of SFI effectors enhances *P. infestans* colonization of *N. benthamiana*. Mean lesion diameter (**A**) and typical disease development symptoms (**B**) are shown for *P. infestans* 7 days post-inoculation over sites on leaves where an effector construct or empty vector was agro-infiltrated 1 day earlier. Each effector was expressed as an N-terminal GFP fusion protein as indicated, except for SFI8. Error bars represent SEM, and significant difference (* = p<0.001) in lesion size compared to empty vector control was determined by one-way ANOVA. Three biological replicates were performed, each using 24 inoculation sites per construct.

The 8 PiRXLR effectors suppressing early MTI signaling in tomato are assumed to contribute significantly to the virulence of *P. infestans*. *N. benthamiana* was further used to explore the role of the 8 selected PiRXLR effectors in host colonization. *Agrobacterium tumefaciens* strains containing the PiRXLR effector construct were infiltrated into leaves of 2–3 week-old *N. benthamiana* plants. Leaves were challenged with *P. infestans* 1 day after agro-infiltration and lesion size (Figure 7A), as well as disease symptoms (Figure 7B), were recorded after an additional 7 days. Except SFI2, whose overexpression in *N. benthamiana* leaves caused cell death that interfered with the pathogen assay, we found that the remaining 7 PiRXLR effectors enhanced colonization of *N. benthamiana* by *P. infestans* (Figure 7A, 7B). Compared to the empty vector control, the expression of the PiRXLR effectors caused a two- to five-fold increase of the lesion size (p<0.001) due to enhanced *P. infestans* colonisation. The strongest effect was observed when expressing GFP-SFI1. Interestingly, this is one of the effectors that localizes predominantly to the nucleus/nucleolus and suppresses flg22-mediated induction of MTI response genes in both Arabidopsis and tomato, but does not suppress MAP kinase activation, suggesting that it may act downstream of this step. We were thus prompted to look further at the significance of the nuclear/nucleolar localization of SFI1 on its virulence function.

### The nuclear localization of SFI1 is required for suppression of MTI signaling in Arabidopsis, and for enhancing *P. infestans* colonization of *N. benthamiana*

**Figure 8:**
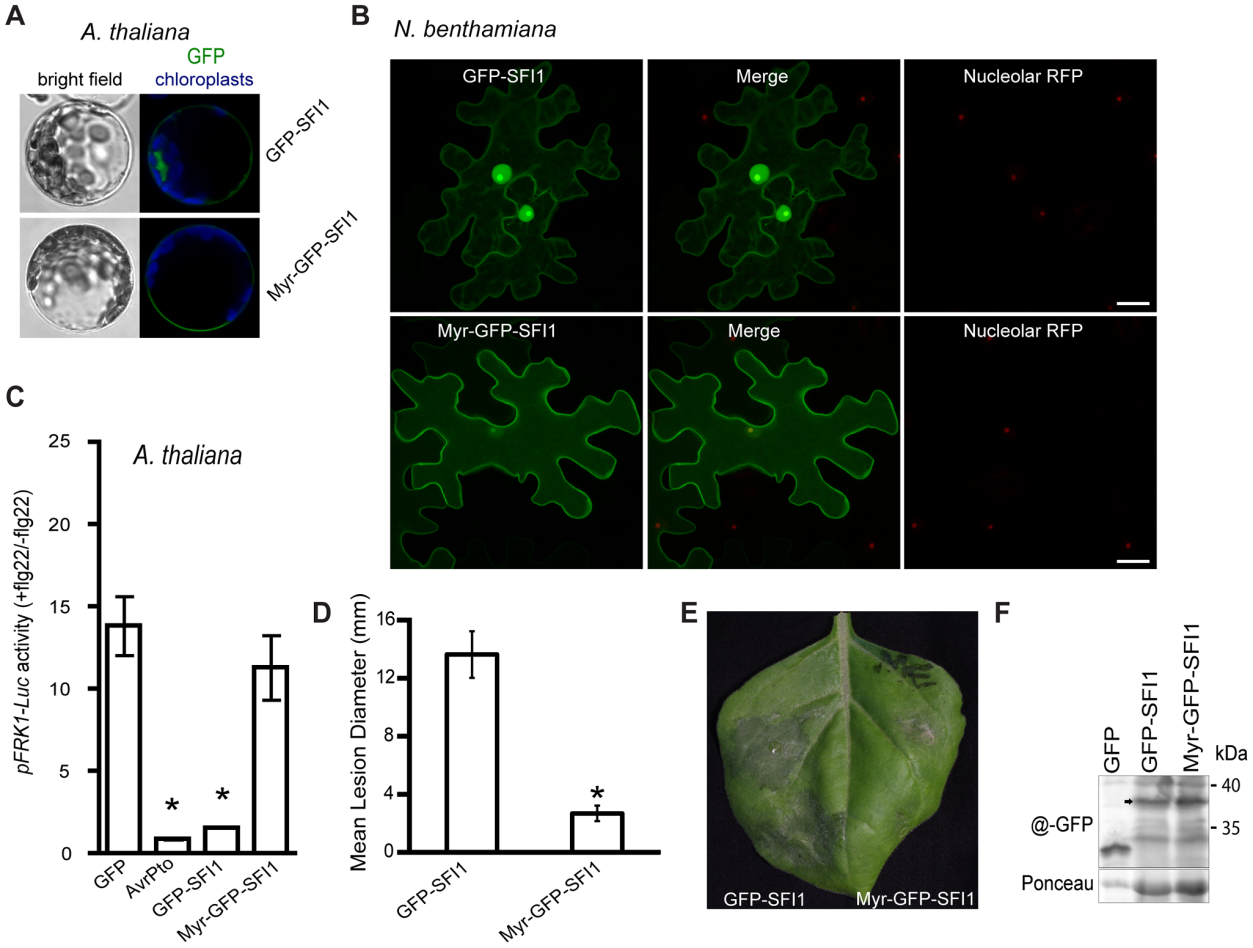
Importance of the nuclear localization of SFI1 for suppression of flg22-triggered *pFRK1-Luc* expression in *A. thaliana* and for *P. infestans* colonization in *N. benthamiana*. (**A**) Confocal microscopy of *A. thaliana* protoplasts expressing GFP-SFI1 or myr-GFP-SFI1 12 h after transfection. Representative optical sections of bright field and merged GFP (in green) and chloroplast (in blue) fluorescence images are shown. (**B**) Representative confocal microscope images of *N. benthamiana* cells expressing GFP-SFI1 and myr-GFP-SFI1 (left panels, in green) with the nucleolar marker RFP-fibrillarin (right panels, in red); the merged images are shown in the central panels. (**C**) Measurement of *pFRK1-Luc* reporter activity in *A. thaliana* protoplasts 6 h after flg22 treatment in the presence of GFP (control), AvrPto, GFP-SFI1 or myr-GFP-SFI1. Pooled data from four experiments are presented as mean ± SEM. Significant differences (p<0.05) in luciferase activity (denoted *) were determined using one-way ANOVA followed by Dunnett's multiple comparison test. (**D, E**) Effect of transient expression of GFP-SFI1 and myr-GFP-SFI1 on *P. infestans* colonization of *N. benthamiana*. Mean lesion diameter (**D**) and typical disease symptoms (**E**) are shown for *P. infestans* 7 days post-inoculation over sites on leaves where an effector construct was agro-infiltrated 1 day earlier. * represents statistical significance (p<0.001) using one-way ANOVA. (**F**) Immunoblot using anti-GFP antibody showing that both GFP-SFI1 and myr-GFP-SFI1 are stable and intact fusion proteins (arrowed) *in planta*.

We attempted to address the importance of the nuclear localization for the function of SFI1 and hypothesized that mis-targeting of the effector away from the nucleus could impact its virulence function. We generated a construct introducing a myristoylation site at the N-terminus of GFP-SFI1. Transient expression of myr-GFP-SFI1 *in planta* showed that the myristoylation site was functional in targeting SFI1 to the plasma membrane in Arabidopsis protoplasts (Figure 8A) and *N. benthamiana* leaves (Figure 8B). Both GFP-SFI1 and myr-GFP-SFI1 fusion proteins were stable and intact *in planta* (Figure 8F). Strikingly, whereas the flg22-dependent induction of *pFRK1-Luc* activity was suppressed by GFP-SFI1 in Arabidopsis protoplasts, no such suppression was observed in the presence of myr-GFP-SFI1 (p-value<0.05 - Figure 8C). Notably, myr-GFP-SFI1 lost its ability to enhance *P. infestans* colonization of *N. benthamiana* (Figure 8D, 8E), providing strong evidence that suppression of MAMP-induced immune responses by this effector in both host and non-host plants requires its localization to the nucleus/nucleolus.

## Discussion

In this study, we used a protoplast-based system to assess the potential for RXLR effectors from *P. infestans* (PiRXLRs) to manipulate MAMP-triggered early signaling in both a host and non-host plant species. Of 33 PiRXLR effector candidates, selected on the basis of up-regulation during the biotrophic phase of late blight infection, 8 (SFI1-SFI8) were able to suppress flg22-mediated induction of *pFRK1-Luc* activity in protoplasts of the host plant tomato (summarized in Table 1). Of these, three (SFI5-SFI7) were shown to suppress flg22-dependent MAP kinase activation at - or upstream of - the step of MAPK- and/or MAPKK kinase activation, indicating that they target the earliest stages of MTI signal transduction in tomato (Table 1). As *P. infestans* does not possess flagellin, the ability of these effectors to attenuate flg22-mediated MAP kinase activation and early defense gene expression indicates that these events are likely stimulated following perception of as yet undefined oomycete MAMPs. We confirmed that 7 of the 8 PiXRLR effectors that suppress early MTI signaling in tomato also enhance colonization by *P. infestans* in the host plant *N. benthamiana* (Table 1).

**Table 1:** Summary of PiRXLR effectors with suppressing activity on MTI.

	Flg22-induced						
PiRXLR	*pFRK1-Luc* activity		MAMP gene expression	MAP kinase activation		Sub-cellular localization	*P. infestans* growth
	*S. lycopersicum*	*A. thaliana*	*A. thaliana*	*S. lycopersicum*	*A. thaliana*	*N. benthamiana*	*N. benthamiana*
SFI1	S	S	S	No	No	nucleus/nucleolus	E
SFI2	S	S	S	No	No	nucleus/nucleolus	n.d
SFI3	S	No	No	No	No	nucleus/nucleolus	E
SFI4	S	No	No	No	No	cytoplasm/nucleus	E
SFI5	S	S	No	S	No	PM	E
SFI6	S	No	No	S	No	cytoplasm/PM	E
SFI7	S	No	No	S	No	cytoplasm/PM	E
SFI8	S	S	S			cytoplasm/nucleus	E
PITG_00821	No	S	No	n.d	n.d	n.d	n.d
PITG_05750	No	S	No	n.d	n.d	n.d	n.d
PITG_16737	No	S	No	n.d	n.d	n.d	n.d
PITG_21388	No	S	No	n.d	n.d	n.d	n.d

S: Suppression No: No suppression E: Enhanced n.d.: not determined.

We found that 3 PiRXLR effectors (SFI1, SFI2 and SFI8/AVRblb2) suppress flg22-mediated induction of *pFRK1-Luc* activity in protoplasts of both the host plant tomato and the non-host plant Arabidopsis. We confirmed that suppression by all 3 effectors attenuates transcriptional activation of endogenous MAMP-induced marker genes in Arabidopsis (Table 1), indicating that some effectors may function efficiently across diverse (host and non-host) plant species. Interestingly, we found another set of 4 PiRXLR effectors that suppressed *pFRK1-Luc* activation only in the non-host Arabidopsis. This was a surprise, albeit the assay is potentially less sensitive in the host plant tomato. However, none of these effectors were able to prevent the activation of endogenous (Arabidopsis) MAMP-induced marker genes (Table 1). Therefore, additional experiments are necessary to determine to what extent suppression of flg22-induced post-transcriptional or translational processes may account for the activity of these effectors on the p*FRK1-Luc* reporter system in this plant.

While 3 PiRXLR effectors (SFI5-SFI7) suppressed MAMP-dependent MAP kinase activation in tomato, no PiRXLR effector had a similar effect in Arabidopsis (Table 1). This is an important finding, consistent with the hypothesis of Schulze-Lefert and Panstruga [63] that non-host resistance in plants (in this case Arabidopsis), which are distantly related to the host of *P. infestans*, is likely to include failures in effector-triggered susceptibility, due to effectors that are not sufficiently adapted to adequately manipulate plant immunity. Each of these observations will be discussed below.

The large number of RXLR effector gene candidates in *Phytophthora* genomes complicates their functional analysis by reverse and forward genetics. Thus, the development of a medium/high- throughput system to explore their function in plants is strongly desired. Other large-scale effector functional screens have been conducted recently. A study by Fabro *et al.* [64] identified 39 out of 64 RXLR effectors from *Hyaloperonospora arabidopsidis* that enhance *P. syringae* growth in Arabidopsis Col-0 when delivered via the type III secretion system (T3SS). A majority of these effectors was additionally able to suppress callose deposition in response to bacterial MAMP perception. Thirteen of the *H. arabidopsidis* RXLR effectors promoted bacterial growth in turnip (*Brassica rapa*), a member of the *Brassicaceae* that is a non-host of *H. arabidopsidis*, indicating that they likely retain their virulence function in this closely related plant. Although the authors did not provide molecular evidence of the influence of these RXLR effectors on MTI in turnip, their results are in line with our conclusions, in that the activity of some RXLR effectors is not restricted to the pathogen's host(s). Nevertheless, a number of *H. arabidopsidis* RXLR effectors that promoted *P. syringae* growth in Arabidopsis either failed to do so (44 effectors) in turnip, suggesting that they fail to function in the non-host plant, or reduced *P. syringae* growth (7 effectors), suggesting that they had activated ETI. Whereas we have identified a set of PiRXLRs that suppress early MTI signaling in tomato but not in Arabidopsis protoplasts, none of the tested PiRXLRs in our study significantly promoted cell death in Arabidopsis protoplasts. In apparent contradiction to the molecular evolutionary concept of non-host resistance [63] we have also identified three PiRXLR effectors that potentially attenuate early flg22-mediated MTI signaling events in Arabidopsis. In order to demonstrate whether failure to suppress MTI has the potential to contribute to non-host resistance to *P. infestans* in Arabidopsis, it would be necessary to extend the analysis to all PiRXLR effectors and provide an in-depth study of their precise function in both host and non-host plant.

Our primary goal was to identify and ascribe functions to PiRXLR effector proteins that interfere with early plant defense responses. Interestingly, AVRblb2 family members (such as SFI8), but not AVR3a, were among effectors suppressing flg22-induced *pFRK1-Luc* activity. This apparently contrasts with the results obtained from the screen for suppression of cell death mediated by the MAMP INF1 in *N. benthamiana*, in which AVR3a but not AVRblb2 family members acted as a suppressor [35], [37], [44]. Similarly, PITG_14736/PexRD8 also suppressed INF1-mediated PCD [44] whilst failing to attenuate flg22-mediated responses in this study, and SFI5/PexRD27 suppressed flg22-mediated MAP kinase activation here, whilst failing to suppress INF1-mediated PCD ([44]; Figure 6). Possible explanations would be that AVR3a and PexRD8 disable components located downstream of the MAMP signal transduction and early transcriptional changes studied here, or that these effectors act specifically on alternative signal transduction events related to INF1-mediated cell death, but not the FLS2/flg22 pathway. The opposite may be true for SFI8/AVRblb2 and SFI5. Moreover, SFI7 suppresses flg22/FLS2-mediated signal transduction and attenuates INF1-mediated PCD, but not Cf-4-mediated PCD, whereas AVR3a attenuates both INF1-mediated and Cf-4-mediated PCD. Evidence is thus emerging of effectors with overlapping functions, at the phenotypic level, that are likely mediated by distinct modes of action at the mechanistic level.

The epistatic analysis of the MAP kinase signaling cascade showed that SFI5-SFI7 presumably act upstream of the activation of the SlMPK1/SIPK and SlMPK3/WIPK MAP kinases in tomato protoplasts following flg22 perception. These effectors potentially function at the FLS2 receptor complex, or upon MAPKKK or MAPKK activity, or upon alternative regulators associated with this signal transduction pathway. As *P. infestans* does not possess flg22, and is thus unlikely to activate FLS2, the activity of any effectors upon the receptor complex must involve targets that are associated with bacterial and oomycete MAMP detection. Nevertheless, the absence of any suppressive activity of these effectors against CF4-mediated cell death and the modest suppression of INF1-mediated PCD only by SFI7 – two defense pathways that utilize alternative MAPKK kinases - imply specificity in the signal transduction pathways targeted by these effectors. It is important to note that all three effectors, to differing degree, associate with the plasma membrane, consistent with a potential action at the level of signal perception. Mukhtar *et al*., [65], postulated that an overlapping subset of host proteins, so-called hubs, are targeted by oomycete (*H. arabidopsidis*) and bacterial (*P. syringae*) effectors that have arisen independently through convergent evolution. Therefore, future work will focus on identification of host proteins with which SFI5-SFI7 interact to better elucidate the molecular mechanisms underlying the action of these effectors.

An effect of AVRblb2 on early MAMP signaling in solanaceous plant species has not been reported before, but it is has been shown that AVRblb2 affects plant immunity by inhibiting the secretion of C14, an apoplastic papain-like cysteine protease [38]. It is worth noting that in that study, AVRblb2 was exclusively localized at the plasma membrane, whereas in our experiments SFI8/AVRblb2 appeared mainly in the nucleus and cytosol. Yet, as AVRblb2 forms a large family and it is not clear which AVRblb2 isoform was exactly tested for the inhibition of C14 secretion [38], any apparent discrepancies in our results raise the possibility that different members of the AVRblb2 family have distinct or multiple cellular activities. Nevertheless, in our study all tested members of the AVRblb2 family were able to significantly suppress flg22-mediated induction of *pFRK1-Luc* activity in protoplasts of the host plant tomato.

As the effectors SFI1, SFI2 and SFI8/AVRblb2 interfere with transcriptional up-regulation of MAMP-responsive genes in both host and non-host plants, we presume that they target conserved processes upstream of the earliest transcriptional responses. None of these effectors prevented MAP kinase activation, suggesting that they act downstream of such signal transduction. The nuclear localization of SFI1 and SFI2 in Arabidopsis, tomato and *N. benthamiana* may indicate that they directly manipulate regulatory processes leading to transcriptional up-regulation. For SFI1 we showed that its mislocalization to the plasma membrane, via addition of a myristoylation signal, prevented both its ability to suppress flg22-mediated MTI gene activation in Arabidopsis and its ability to enhance *P. infestans* colonization of the host plant *N. benthamiana.* This strongly implicates the nucleus as the site of effector activity for SFI1. It also indicates the importance of determining subcellular localization of effectors, as mis-targeting them provides a strategy for investigating their virulence function. The fact that SFI1 activity is apparently conserved in the non-host plant Arabidopsis indicates that we may draw on the wealth of genetic resources available in the model plant to further dissect the functions of this effector. Future work will employ additional mis-targeting approaches, for example nuclear export (NES) and nuclear localization (NLS) signals, to better elucidate the potential contributions of SFI1-SFI8 activities, either within or outside the nucleus, to suppress early MTI signaling.

Three of the PiRXLR effectors, SFI5-SFI7, suppressed flg22-mediated post-translational MAP kinase activation in tomato but not in the non-host Arabidopsis. A further two effectors, SFI3 and SFI4, were shown to suppress specifically *pFRK1-Luc* activation in tomato, although we need to confirm their inhibitory effect on the expression of endogenous MAMP-responsive genes. Nevertheless, each enhanced *P. infestans* colonization when transiently expressed in *N. benthamiana*, consistent with a role in MTI suppression. Functional characterization of all these effectors is thus better pursued in host plants within the *Solanaceae*. The availability of genome sequences for potato [66], tomato [67] and *N. benthamiana* [68], the genetic tractability of the diploid tomato [67], and the range of functional analyses that can be performed in *N. benthamiana* [52], considerably broaden opportunities to do this. Moreover, the adaptation of the Arabidopsis protoplast-based system [48], [49], [58] to investigate the earliest stages of MTI in tomato, presented here, further enhances capabilities to study the functions of effectors from pathogens that infect solanaceous hosts. Future work will employ transgenic host and nonhost plants expressing the effectors revealed here, and additional RXLR effectors from *P. infestans*, to more specifically investigate their precise mechanistic action. Such studies will also reveal those effectors which may act downstream of the earliest signaling events in order to suppress MAMP-triggered immunity.

Ectopic expression in *N. benthamiana* of 7 of the 8 SFI effectors selected through the protoplast-based screen enhanced plant susceptibility toward *P. infestans* infection. This result suggests that the suppression of early signaling events triggering basal immunity is an important step toward successful host colonization by this pathogen. *P. infestans* itself offers the possibility to further study functional aspects of PiRXLR effectors, and gain- and loss-of function experiments may confirm the importance of our candidate effectors for virulence. However, it should be noted that the functional redundancy of the PiRXLR effectors studied here in suppressing early FLS2/flg22 MTI signaling suggests that silencing of these effector genes in *P. infestans* may not lead to clear virulence phenotypes, as has been shown by deletion studies with type III effectors in *P. syringae* [69]. Nevertheless, silencing of single PiRXLR effector genes *Avr3a* [36], or *PITG_03192* [43] compromised *P. infestans* pathogenicity, indicating that (at least some of) the functions of these effectors are not redundant.

In conclusion, the tomato protoplast system provides a new medium/high-throughput tool to identify effectors that modulate the earliest stages of MTI signal transduction. We have identified 8 PiRXLR effectors that suppress early flg22-mediated MTI in tomato. Three of these reveal association with the plant plasma membrane and act at, or upstream of, MAPKK activation specifically related to flg22-mediated MTI signal transduction. Two of these effectors, SFI5 and SFI6, apparently do not act on other MAP kinase-mediated signal transduction events studied in this investigation. In addition, five of the effectors act downstream of the MAP kinase cascade, 3 of which also clearly suppress early flg22-mediated gene induction in Arabidopsis. This demonstrates that the effector complement of *P. infestans* contains functional redundancy in the context of suppressing early MTI signal transduction and gene activation. It remains to be established why such functional redundancy is necessary, or is selected for, and it is consistent with studies of bacteria such as *P. syringae* [69] that plant pathogens evolve multiple means of confounding the host immune system.

## Materials and Methods

### Plant growth conditions

*Solanum lycopersicum* cv. Moneymaker was kept in a greenhouse under controlled growth conditions: 16 h light at 24°C/8 h dark at 22°C, 40%–45% humidity, ∼200 µE m^−2^ s^−1^ light intensity. They were grown on soil containing a 4.6∶4.6∶1 mixture of type P soil, type T soil (Patzer, Germany) and sand. Leaves from 3 to 4 week-old plants were used for experiments.

*Arabidopsis thaliana* plants of the Col-0 ecotype were cultivated in a phytochamber under stable climate conditions: 8 h light at 22–24°C/16 h dark at 20°C, 40%–60% humidity, ∼120 µE m^−2^ s^−1^ light intensity. They were grown on soil composed of a 3.5∶1 mixture of GS/90 (Patzer, Germany) and vermiculite. Leaves from 4 to 5 week-old plants were used for protoplast preparation.

*Nicotiana benthamiana* was grown as described previously [36].

### *Phytophthora infestans* RXLR effector cloning

*Phytophthora infestans* putative RXLR effector genes (PiRXLR) were amplified minus the signal peptide from gDNA of the sequenced isolate T30-4 in a two-step nested PCR reaction in order to add flanking attB sites to the RXLR coding sequence. The cloning primers are shown in Table S1 and Table S2. The PCR products were recombined into pDONR201 or pDONR221 vectors (Invitrogen) to generate entry clones, which were further recombined into the vectors p2GW7, p2FGW7 (N-terminal GFP fusion), pB7WGF2 (N-terminal GFP fusion), p2GWF7 (C-terminal GFP fusion) or p2HAGW7 (N-terminal hemagglutinin-tag; derived from p2GW7) (VIB, Ghent University, Belgium) using the Gateway recombination cloning technology (Invitrogen). The myristoylation signal sequence MGCSVSK was added to the amino-termini of the GFP-PiRXLR fusions using PCR with modifying primers and restriction cloning into pENTR1a (Invitrogen) before recombination into p2GW7 or pB2GW7 (VIB, Ghent University, Belgium). The Gateway destination vectors used are designed for transient 35S promoter-driven gene expression in protoplasts or, in the case of pB7WGF2 and pB2GW7, in *N. benthamiana* plants.

### *S. lycopersicum* MAPK kinase and MAPKK kinase cloning

To generate the constructs used for epistasic analysis of the MAP kinase signaling cascade, four primer combinations: *SlMEK1-attB1/SlMEK1-attB2*, *SlMEK2-attB1/SlMEK2-attB2*, *SlMAP3Kα-attB1/SlMAP3Kα-attB2* and *SlMAP3Kε-attB1/SlMAP3Kε-attB2* (listed in Table S3) were used to amplify by PCR *SlMEK1-DD*, *SlMEK2-DD*, *SlMAP3Kα-KD* and *SlMAP3Kε-KD* from pER8 plasmid constructs, respectively. Subsequently, Gateway attB linkers were added via PCR using primers *attB1-adapter* and *attB2-adapter*. The obtained PCR products were introduced into pDONR201 to generate entry clones using the Gateway recombination cloning technology (Invitrogen). The genes were further recombined into the vector p2GWF7 (C-terminal GFP fusion – VIB, Ghent University, Belgium). The resulting plasmid constructs, *p35S-SlMEK1-DD-GFP*, *p35S-SlMEK2-DD-GFP*, *p35S-SlMAP3Kα-KD-GFP* and *p35S-SlMAP3Kε-KD-GFP* were used for protoplast transfection as described below.

### Plasmid DNA preparation

Plasmid DNA was isolated from *E. coli* DH5α liquid cultures by column purification using the PureYield Plasmid Midi-prep system (Promega) following the manufacturer's instructions. For selected candidate gene, control genes and reporter gene constructs, higher amount of the corresponding plasmids were purified on a cesium chloride density gradient.

### Protoplast preparation and transfection

*S. lycopersicum* mesophyll protoplasts were prepared as described by Nguyen *et al*., [52] with slight modifications. The lower epidermis of fully expended leaflets was gently rubbed with grated quartz, rinsed with sterile water and leaf strips were floated on enzyme solution containing 2% cellulose ‘Onozuka’ R10 (Yakult Pharmaceutical Industry), 0.4% pectinase (Sigma) and 0.4 M sucrose in K3 medium. After 30 min vacuum-infiltration and 3 h incubation at 30°C in the dark, the enzyme-protoplast mixture was filtered through a 45–100 µm nylon mesh. Viable protoplasts were collected by sucrose gradient centrifugation and washed in W5 buffer. After recovery on ice for 2 h, protoplasts were harvested by centrifugation and suspended to a density of 6*10^5^ cells/ml in MMG buffer prior polyethylene glycol-mediated transfection. 100 µg plasmid DNA/ml protoplast suspension was used during transfection. Protoplasts samples were then incubated in W1 buffer at 20°C in the dark for 12 to 16 h allowing plasmid gene expression.

The preparation of *A. thaliana* mesophyll protoplasts was performed according to the protocol from Yoo *et al*., [70] with minor changes. Briefly, thin leaf stripes were dipped into 1.5% cellulose ‘Onozuka’ R10 – 0.4% macerozyme R10 solution (Yakult Pharmaceutical Industry), vacuum-infiltrated for 30 min and digested for 3 h at 20°C in the dark. After two subsequent washing steps with W5 buffer Arabidopsis protoplasts were suspended in MMG buffer to a concentration of 2*10^5^ cells/ml. Arabidopsis protoplast transfection was performed as for tomato.

### Luciferase and β-glucuronidase (GUS) reporter gene assays

Luciferase and GUS reporter gene assays were conducted to screen for immunity-suppressing effector genes. For this, *A. thaliana* or *S. lycopersicum* protoplasts were co-transfected with *pFRK1-Luc*, *pUBQ10-GUS* and an effector gene construct (or empty p2FGW7 serving as GFP control). For the luciferase assay, luciferin was added to 600 µl transfected protoplast solution to a final concentration of 200 µM. Protoplasts were transferred to an opaque 96-well plate (100 µl per well). For each sample, flg22 was added to 3 wells to a final concentration of 500 nM (+flg22). The remaining 3 replicates were left untreated (−flg22). The luminescence reflecting the luciferase activity was measured at different time-points using a Berthold Mithras LB 940 luminometer. For the GUS assay, 50 µl transfected protoplast solution of each sample was treated with 500 nM flg22 (+flg22) and 50 µl were left untreated (−flg22). Protoplast pellets were collected 3 or 6 h after flg22 elicitation. The cells were lysed in 100 µl CCLR solution (cell culture lysis reagent, Promega). For each sample, 3 technical replicates of 10 µl were incubated with 90 µl MUG substrate (1 mM 4-methyl-umbelliferyl-β-D-glucuronide, 100 mM Tris-HCl pH 8.0, 2 mM MgCl_2_) for 30 min at 37°C. The reaction was stopped with 900 µl 0.2 M Na_2_CO_3_. The fluorescence was monitored in an opaque plate using a MWG 96-well plate reader with λ_ex_ = 360 nm and λ_em_ = 460 nm.

### Statistical analysis of reporter gene assay data

Raw data of Luciferase and GUS assays were processed using Microsoft Excel. First the mean value of the +flg22 and the −flg22 triplicates was calculated for each sample in both assays of an experiment. Next, the +flg22/−flg22 ratio was calculated using the values from the 3 or 6 h time-point of the Luciferase assay and divided by the corresponding +flg22/−flg22 ratio of the GUS assay for normalization. Statistical analysis was performed using one-way ANOVA followed by Dunnett's multiple comparison test.

### RNA isolation, cDNA synthesis and quantitative real-time PCR (qRT-PCR)

Total RNA from 400 µl *A. thaliana* protoplasts was extracted with TRI reagent (Ambion) and treated with DNase I (Machery-Nagel) following the suppliers' protocols. Poly A-tailed RNA (1 µg) was converted to cDNA using the RevertAid reverse transcriptase (Fermentas) and oligo-dT primers. qRT-PCR reactions were performed in triplicates with Maxtra SYBR Green Master Mix (Fermentas) and were run on a Biorad iCycler according to the manufacturers' instructions. Relative gene expression was determined with a serial cDNA dilution standard curve. The *Actin* transcript was used as an internal control in all experiments. Data was processed with the iQ software (Biorad).

qRT-PCR to measure PiRXLR gene expression was carried out on a time-course of potato leaves (cv Desiree) infected with *P. infestans* isolate 88069. Total RNA from infected leaf discs was extracted with RNeasy Plant mini kit (Qiagen) and treated with DNase I (Qiagen) following the suppliers' protocols. Poly A-tailed RNA (1 µg) was converted to cDNA using the Superscript II reverse transcriptase (Invitrogen) and oligo-dT primers. qRT-PCR reactions were performed in triplicate with Power SYBR Green Master Mix (ABgene) and run on a Biorad Chromo4 cycler according to the manufacturer's instructions. Relative gene expression was determined using the ΔΔCT method, and *P. infestans ActA* gene was used as an internal control in all experiments, as described in Whisson *et al* [32]. Data was processed with Opticon monitor software (Biorad). Primers used in qRT-PCR reactions are listed in Table S3.

### Detection of phosphorylated MAP kinase and GFP-fusion proteins by immunoblotting

To monitor the activation of MAP kinase, transfected protoplasts were challenged with 500 nM flg22. Pellets from 100 µl protoplast solution were collected 0, 15 and 30 min after treatment and denatured in protein loading buffer. Proteins were loaded onto a 13.5% SDS-polyacrylamid gel and separated by electrophoresis (SDS-PAGE) using the Biorad MiniProtean equipment according to the manufacturer's instructions. PageRuler Prestained protein ladder (Fermentas) was used as a molecular weight marker. Proteins were blotted onto nitrocellulose membranes (Hybond–ECL, Amersham) and stained with 0.1% Ponceau S to visualize equal sample loading. The membranes were blocked for 1 h at room temperature in 5% skimmed milk in TBS-T buffer (50 mM Tris-HCl pH 7.5, 150 mM NaCl, 0.1% Tween 20), incubated overnight at 4°C in primary antibody solution (anti-phospho-p44/42 MAPK antibody, dilution 1/1000 in 5% BSA in TBS-T, Cell Signaling Technology) and finally incubated for 1 h at room temperature in secondary antibody solution (alkaline phosphatase-coupled anti-rabbit IgG antibody, dilution 1/3000 in TBS-T, Sigma). The immunoblot was revealed in NBT/BCIP detection solution.

The expression of GFP-tagged PiRXLR effectors was assessed by immunoblotting using polyclonal anti-GFP antibody produced in rabbit or in goat (Acris Antibodies) at a 1/3000 dilution in 5% BSA in TBS-T. For this, protoplast samples were collected 12 (for *S. lycopersicum*) or 24 h (for *A. thaliana*) after transfection and SDS-PAGE and immunoblotting were carried out as described above.

### Immunoprecipitation and *in vitro* kinase assay

The MAP kinase *in vitro* kinase assay was carried out as described by He *et al*., [48]. Briefly, 1 ml transfected protoplasts were lysed in 1 ml of immunoprecipitation (IP) buffer (150 mM NaCl, 50 mM HEPES pH 7.4, 1 mM EDTA, 1 mM DTT, 0,1% Triton X-100, 1× phosphatase inhibitor cocktail [PhosphoSTOP, Roche Applied Science] and 1× protease inhibitor cocktail [Complete EDTA-free, Roche Applied Science]). HA-tagged SlMPK1 and SlMPK3 kinases [52] were immunoprecipitated from lysates after adding 20 µl anti-H antibody-coupled beads (Roche Applied Science) and incubated for 3 h at 4°C with gentle shaking. After centrifugation at 500 g for 1 min, the immunoprecipitated material was washed with IP buffer followed by a wash with kinase buffer (20 mM Tris-HCl pH 7.5, 20 mM MgCl_2_, 5 mM EDTA and 1 mM DTT). The kinase reaction was performed by adding 25 µl of kinase buffer (0.25 mg/ml MBP, 100 µM ATP and 5 µCi [γ-^32^P] ATP) for 30 min at RT. The reaction was stopped with 4× SDS-PAGE loading buffer. The ^32^P-labeled MBP was separated by SDS/PAGE (15%) gel and visualized by autoradiography.

### Cell death and sub-cellular localization studies

To determine the cell death rate after transfection (percentage of dead cells/total number of cells), 100 µl protoplast samples were incubated for 24 h and subsequently stained with 1 µg propidium iodide. Stained protoplasts were counted using a Nikon Eclipse 80i epifluorescence microscope with the following filter: TRITC EX 540/40, DM 565, BA 605/55. For sub-cellular localization studies protoplasts were monitored 12 h post-transfection and *N. benthamiana* leaves at 2 days post-infiltration. Imaging was performed using Leica TCS SP2 AOBS confocal microscopes with HCX PL APO lbd.BL 63×1.20 W, L 40×0.8 and L 20×0.5 water immersion objectives. Samples were excited by an argon laser and fluorescence emission was detected at 496–552 nm for GFP and 620–726 nm for chloroplasts. The pinhole was set to 1.5 airy units for protoplasts and 1 airy unit for leaf cells. Single optical section images were acquired from protoplasts and z-stacks were collected from leaf cells and projected and processed using the Leica LCS software and Adobe Photoshop CS3.

### *Agrobacterium*-mediated effector expression

*A. tumefaciens* transformed with pB7WG2 or pB7WGF2 vector constructs were grown overnight, pelleted, re-suspended in infiltration buffer (10 mM MES pH 5.6, 10 mM MgCl_2_ and 200 µM acetosyringone) and adjusted to the required OD_600_ before infiltration into *N. benthamiana* leaves.

### Cell death suppression

*A. tumefaciens* cultures were grown as above and subsequently mixed together to a final optical density at 600 nm (OD_600_) of 0.3 for each construct except Cf4, which was used at 0.6, *N. benthamiana* plants were infiltrated using a 1 ml needleless syringe through the lower leaf surface. Three leaves on six plants were used for each biological replicate. Cell death was scored at 7 d post-infiltration (dpi). An individual inoculation was counted as positive if >50% of the inoculated area developed clear cell death. The mean percentage of total inoculations per plant developing cell death of combined data from at least two biological replicates was calculated. One-way ANOVA was performed to identify statistically significant differences.

### *P. infestans* infection assay

*A. tumefaciens* Transient Assays (ATTA) in combination with *P. infestans* infection were carried out as described [36]. Briefly, *Agrobacterium* cultures were re-suspended in infiltration buffer at a final concentration of OD_600_ = 0.1 and infiltrated in *N. benthamiana* with the bacteria harboring the vector control on one side of the leaf midrib and the bacteria harboring the PiRXLR effector constructs to be tested on the other. *P. infestans* strain 88069 cultured on Rye Agar at 19°C for 2 weeks was used for plant infection. Plates were flooded with 5 ml cold H_2_O and scraped with a glass rod to release sporangia. The resulting solution was collected and sporangia numbers were counted using a haemocytometer and adjusted to 30,000 sporangia/ml. After 1 day, each agro-infiltration site was inoculated with 10 µl droplets of sporangia. Three leaves per plant for 4–6 intact plants were used for each biological replicate. Lesions were measured and photographed at 7 days post-infection and data of at least two biological replicates were combined. Statistically significant differences in lesion size were identified by one-way ANOVA with pairwise comparisons performed using the Holm-Sidak method.

## Supporting Information

Figure S1 *S. lycopersicum* and *A. thaliana* protoplasts used as transient expression systems for reporter gene assays and monitoring of MAP kinase activation. (**A, B**) Mesophyll *A. thaliana* (**A**) or *S. lycopersicum* (**B**) protoplasts were co-transfected with the two reporter gene constructs *pFRK1-Luc* and *pUBQ10-GUS* and either *p35S-GFP* (control vector), *p35S-AvrPto-GFP* (*P. syringae* effector AvrPto) or *p35S-AvrPto G2A-GFP* (non-myristoylated AvrPto). Protoplasts were treated with flg22 (+flg22) or left untreated (−flg22) and reporter gene activities were assayed 3 or 6 h later for *S. lycopersicum* and *A. thaliana*, respectively. For each data set, flg22-induced luciferase activity was calculated relative to the untreated sample and was normalized by the corresponding GUS activities in flg22 and untreated sample (*pFRK1-Luc* activity +flg22/−flg22). Seven independent biological experiments were carried out. Within each experiment three technical replicates were performed. Pooled data are presented as mean ± SEM. One-way ANOVA followed by Dunnett's multiple comparison test was used to decipher statistically significant differences in luciferase/GUS activity between GFP-expressing and *P. syringae* effector expressing protoplasts. An asterisk marks data sets with a p-value<0.05. (**C, D**) MAP kinase activation upon flg22 challenge in *A. thaliana* (**C**) and *S. lycopersicum* (**D**) protoplasts. Immunoblotting of phosphorylated MAP kinase was performed with GFP-, AvrPto-GFP- or AvrPto G2A-GFP-producing protoplast samples collected 0, 15 and 30 min after flg22 treatment. A cross-reacting antibody raised against phosphorylated mammalian MAP kinase p44/p42 was used for detection. GFP and GFP fusion protein presence was confirmed for the same sample set using an anti-GFP antibody. The experiment is representative of at least two repeats. Ponceau S staining served as a control for equal sample loading (RuBisCO signal shown).
(TIF)

Figure S2 Cell death rate in *S. lycopersicum* protoplasts transiently producing N-terminally GFP-tagged SFI effectors. (**A**) Dead cells were stained with propidium iodide (PI) 24 h after transfection with *p35S-GFP* control and observed with epifluorescence microscopy. (**B**) The number of dead and the total number of protoplasts were assessed to determine the percentage of cell death. Three independent experiments were performed where at least 150 protoplasts were counted per data set. Mean values ± SEM are presented. One-way ANOVA followed by Dunnett's multiple comparison test was performed to statistically compare the *p35S-GFP-effector*-transfected protoplasts to the *p35S-GFP* control. ns = non-significant.
(TIF)

Figure S3 Luciferase reporter gene assay in protoplasts expressing *P. infestans* AVRblb2 family members. (**A, B**) Mesophyll protoplasts from *S. lycopersicum* (**A**) and *A. thaliana* (**B**) were used and experiments and statistical analysis were carried out as described in Figure S1. Mean values ± SEM are from four independent experiments.
(TIF)

Figure S4 Expression profiles of SFI effector genes during a time-course of potato infection. The expression of SFI genes was assessed across time-points after potato (cv Desiree) inoculation (24–60 hpi) relative to their expression in sporangia (S), which was given a value of 1. Expression of each gene was normalized against the endogenous *P. infestans ActA* gene. Each expression point is the combined analysis from 3 biological replicates and error bars represent ± SEM.
(TIF)

Figure S5 Cell death rate in *A. thaliana* protoplasts transiently producing N-terminally GFP-tagged SFI effectors. (**A**) Dead cells were stained with propidium iodide (PI) 24 h after transfection with *p35S-GFP* control. (**B**) The number of dead and the total number of protoplasts were assessed to determine the percentage of cell death. Three independent experiments were performed where at least 150 protoplasts were counted per data set. Mean values ± SEM are presented. One-way ANOVA followed by Dunnett's multiple comparison test was performed to statistically compare the *p35S-GFP-effector*-transfected protoplasts to the *p35S-GFP* control. ns = non-significant.
(TIF)

Figure S6 Expression profile of N-terminally GFP-tagged SFI effectors in protoplasts (**A, B**) and *N. benthamiana* leaves (**C**). Immunoblotting with anti-GFP antibody was carried out on protoplast samples from *S. lycopersicum* (**A**) and *A. thaliana* (**B**) 24 h post-transfection and on *N. benthamiana* (**C**) leaf extracts 48 h post-inoculation with *A. tumefaciens*. Signals corresponding to the different GFP fusion proteins are pointed out with an arrow. The asterisk indicates a non-specific signal. All effectors have the expected apparent molecular weight. Partial protein degradation was observed in some samples. The experiment is representative of two to three repeats. Ponceau S staining served as a loading control.
(TIF)

Figure S7 Luciferase reporter gene assay in protoplasts expressing N-terminally GFP-tagged SFI effectors. (**A, B**) Mesophyll protoplasts from *S. lycopersicum* (**A**) and *A. thaliana* (**B**) were used and experiments and statistical analysis were carried out as described in Figure S1. Mean values ± SEM are from at least three independent experiments.
(TIF)

Figure S8 Luciferase reporter gene assay in protoplasts expressing the N- and C-terminally GFP-tagged SFI8/AVRblb2 (**A, B**). Mesophyll protoplasts from (**A**) *S. lycopersicum* and (**B**) *A. thaliana* were used and experiments and statistical analysis were carried out as described in Figure S1. Mean values ± SEM are from at least three independent experiments.
(TIF)

Figure S9 Sub-nuclear localization in *N. benthamiana* of SFI effectors. Typical confocal microscope close-up images of nuclei in *N. benthamiana* leaf cells expressing free GFP (GFP) as a control and N-terminally GFP-tagged SFI effectors (SFI numbers indicated).
(TIF)

Figure S10 MAP kinase *in vitro* kinase assay in *S. lycopersicum* protoplasts. (**A**) GFP or constitutively active MAPK kinase with C-terminal GFP tag (SlMEK1-DD-GFP and SlMEK2-DD-GFP) were co-expressed with hemagglutinin (HA)-tagged *S. lycopersicum* MAP kinase SlMPK1 or SlMPK3. (**B**) GFP or the active kinase domain of MAPKK kinase with C-terminal GFP tag (SlMAP3Kα-KD-GFP and SlMAP3Kε-KD-GFP) were co-expressed with hemagglutinin (HA)-tagged *S. lycopersicum* MAP kinase SlMPK1 or SlMPK3. (**A, B**) HA-tagged MAP kinase were immunoprecipitated with anti-HA antibody for an *in vitro* kinase assay with [γ-^32^P] ATP and myelin basic protein as phosphorylation substrate (MBP^32^P - upper panels). Endogenous MAP kinase activation was detected with antibody raised against activated MAP kinase p44/p42 (middle panels). The lower panels present immunoblots with anti-HA and anti-GFP antibodies showing the expression of HA- and GFP-tagged proteins, respectively. Coomassie blue staining served as a loading control. The experiments are representative of at least two repeats.
(TIF)

Table S1 List of the PiRXLR effector genes tested in the MTI-suppressor screen in *S. lycopersicum* and *A.thaliana* protoplasts. Gene identification number, affiliation to an RXLR gene family [28], nucleotide and protein sequence (without signal peptide) are presented.
(XLSX)

Table S2 List of the PiRXLR effector genes of the AVRblb2 family that were tested in the MTI-suppressor screen in *S. lycopersicum* and *A.thaliana* protoplasts. Gene identification number, affiliation to an RXLR gene family [28], [36], nucleotide and protein sequence (without signal peptide) are presented.
(XLSX)

Table S3 List of used primer sequences.
(XLSX)
