# Supplementary figures and images for: Functionally Redundant RXLR Effectors from Phytophthora infestans Act at Different Steps to Suppress Early flg22-Triggered Immunity
Source: PLoS Pathog. 2014 Apr 24;10(4):e1004057. doi: 10.1371/journal.ppat.1004057 (PMC3999189; doi:10.1371/journal.ppat.1004057)

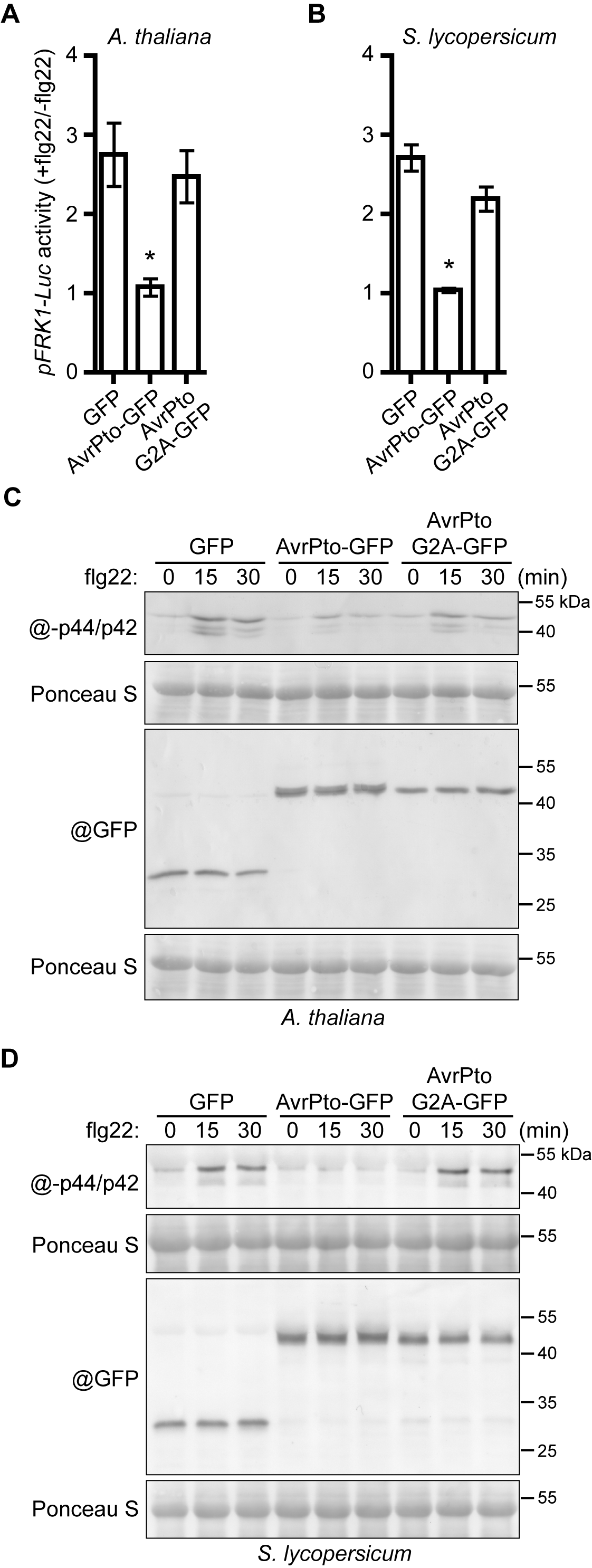

Supplement: Figure S1 — S. lycopersicum and A. thaliana protoplasts used as transient expression systems for reporter gene assays and monitoring of MAP kinase activation. (A, B) Mesophyll A. thaliana (A) or S. lycopersicum (B) protoplasts were co-transfected with the two reporter gene constructs pFRK1-Luc and pUBQ10-GUS and either p35S-GFP (control vector), p35S-AvrPto-GFP (P. syringae effector AvrPto) or p35S-AvrPto G2A-GFP (non-myristoylated AvrPto). Protoplasts were treated with flg22 (+flg22) or left untreated (−flg22) and reporter gene activities were assayed 3 or 6 h later for S. lycopersicum and A. thaliana, respectively. For each data set, flg22-induced luciferase activity was calculated relative to the untreated sample and was normalized by the corresponding GUS activities in flg22 and untreated sample (pFRK1-Luc activity +flg22/−flg22). Seven independent biological experiments were carried out. Within each experiment three technical replicates were performed. Pooled data are presented as mean ± SEM. One-way ANOVA followed by Dunnett's multiple comparison test was used to decipher statistically significant differences in luciferase/GUS activity between GFP-expressing and P. syringae effector expressing protoplasts. An asterisk marks data sets with a p-value<0.05. (C, D) MAP kinase activation upon flg22 challenge in A. thaliana (C) and S. lycopersicum (D) protoplasts. Immunoblotting of phosphorylated MAP kinase was performed with GFP-, AvrPto-GFP- or AvrPto G2A-GFP-producing protoplast samples collected 0, 15 and 30 min after flg22 treatment. A cross-reacting antibody raised against phosphorylated mammalian MAP kinase p44/p42 was used for detection. GFP and GFP fusion protein presence was confirmed for the same sample set using an anti-GFP antibody. The experiment is representative of at least two repeats. Ponceau S staining served as a control for equal sample loading (RuBisCO signal shown). (TIF) [file ppat.1004057.s001.tif]

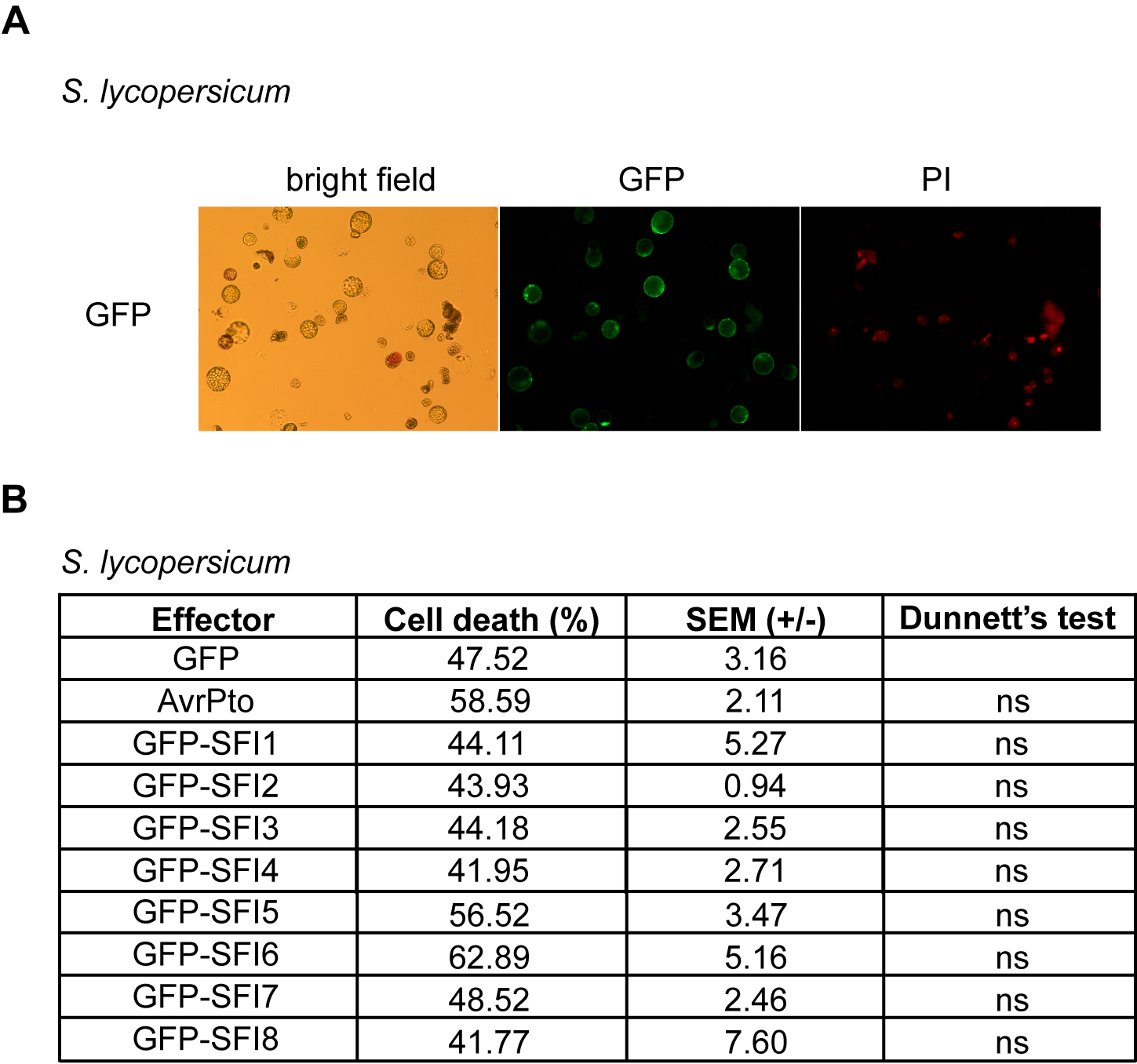

Supplement: Figure S2 — Cell death rate in S. lycopersicum protoplasts transiently producing N-terminally GFP-tagged SFI effectors. (A) Dead cells were stained with propidium iodide (PI) 24 h after transfection with p35S-GFP control and observed with epifluorescence microscopy. (B) The number of dead and the total number of protoplasts were assessed to determine the percentage of cell death. Three independent experiments were performed where at least 150 protoplasts were counted per data set. Mean values ± SEM are presented. One-way ANOVA followed by Dunnett's multiple comparison test was performed to statistically compare the p35S-GFP-effector-transfected protoplasts to the p35S-GFP control. ns = non-significant. (TIF) [file ppat.1004057.s002.tif]

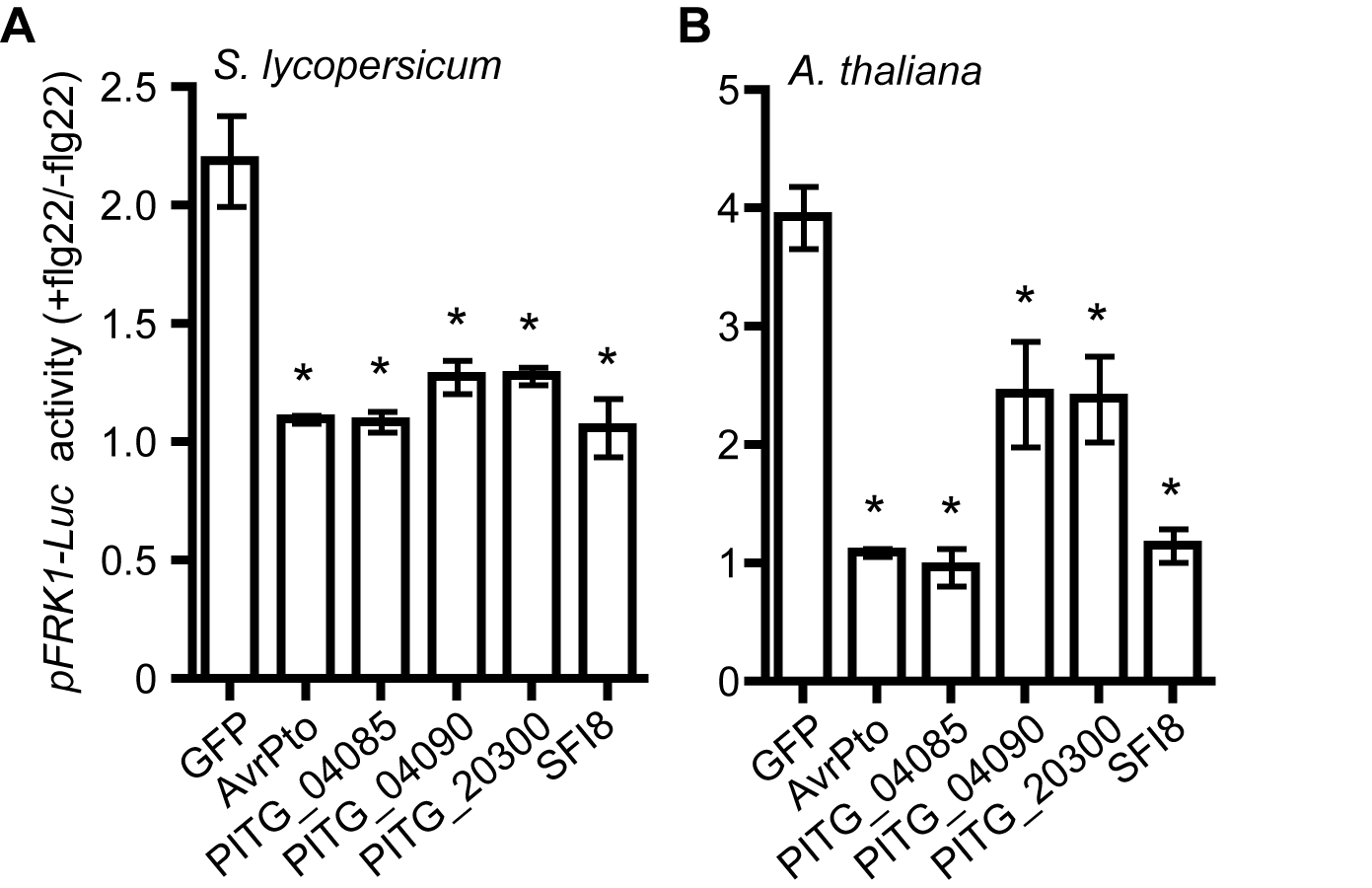

Supplement: Figure S3 — Luciferase reporter gene assay in protoplasts expressing P. infestans AVRblb2 family members. (A, B) Mesophyll protoplasts from S. lycopersicum (A) and A. thaliana (B) were used and experiments and statistical analysis were carried out as described in Figure S1. Mean values ± SEM are from four independent experiments. (TIF) [file ppat.1004057.s003.tif]

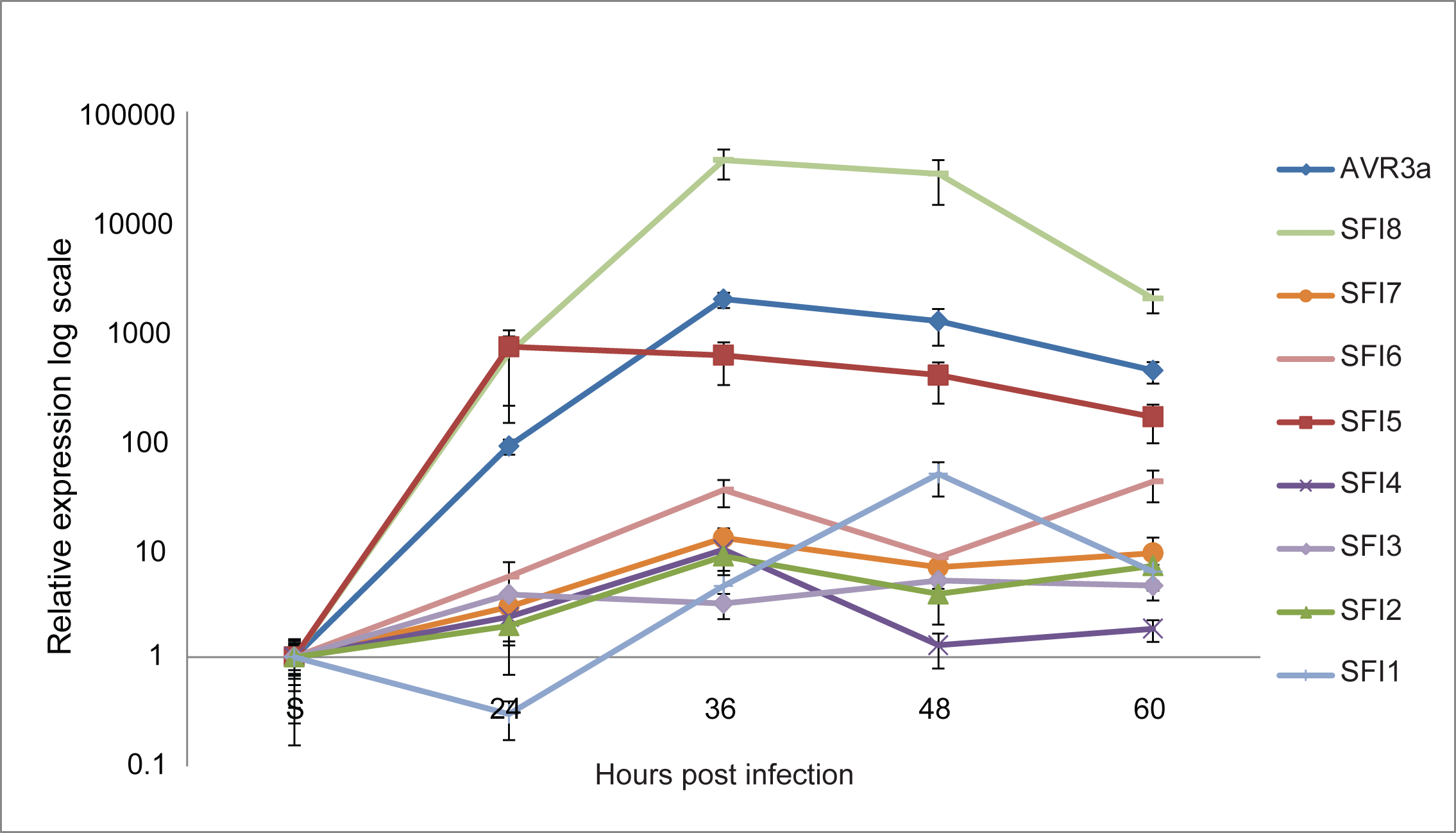

Supplement: Figure S4 — Expression profiles of SFI effector genes during a time-course of potato infection. The expression of SFI genes was assessed across time-points after potato (cv Desiree) inoculation (24–60 hpi) relative to their expression in sporangia (S), which was given a value of 1. Expression of each gene was normalized against the endogenous P. infestans ActA gene. Each expression point is the combined analysis from 3 biological replicates and error bars represent ± SEM. (TIF) [file ppat.1004057.s004.tif]

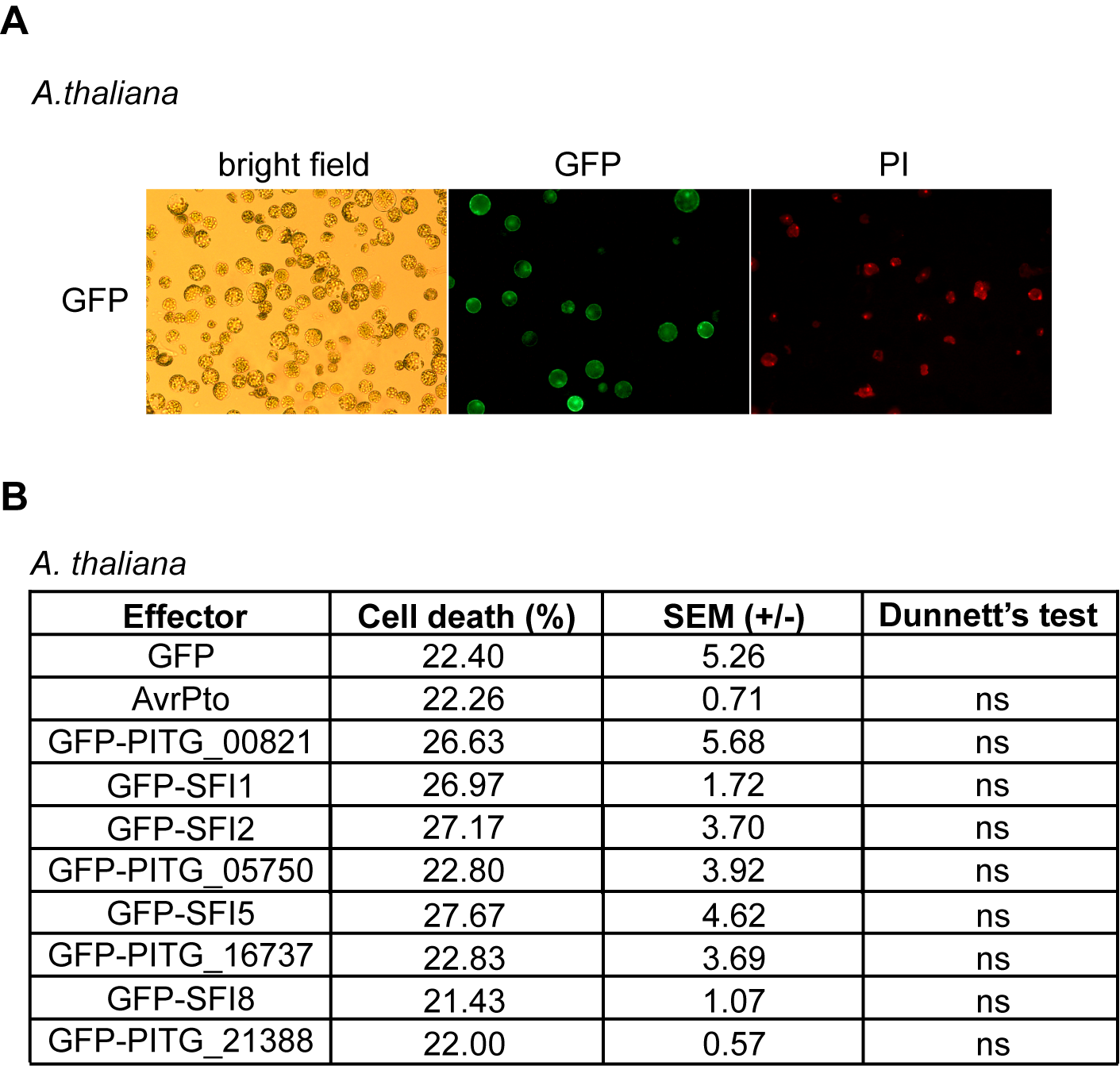

Supplement: Figure S5 — Cell death rate in A. thaliana protoplasts transiently producing N-terminally GFP-tagged SFI effectors. (A) Dead cells were stained with propidium iodide (PI) 24 h after transfection with p35S-GFP control. (B) The number of dead and the total number of protoplasts were assessed to determine the percentage of cell death. Three independent experiments were performed where at least 150 protoplasts were counted per data set. Mean values ± SEM are presented. One-way ANOVA followed by Dunnett's multiple comparison test was performed to statistically compare the p35S-GFP-effector-transfected protoplasts to the p35S-GFP control. ns = non-significant. (TIF) [file ppat.1004057.s005.tif]

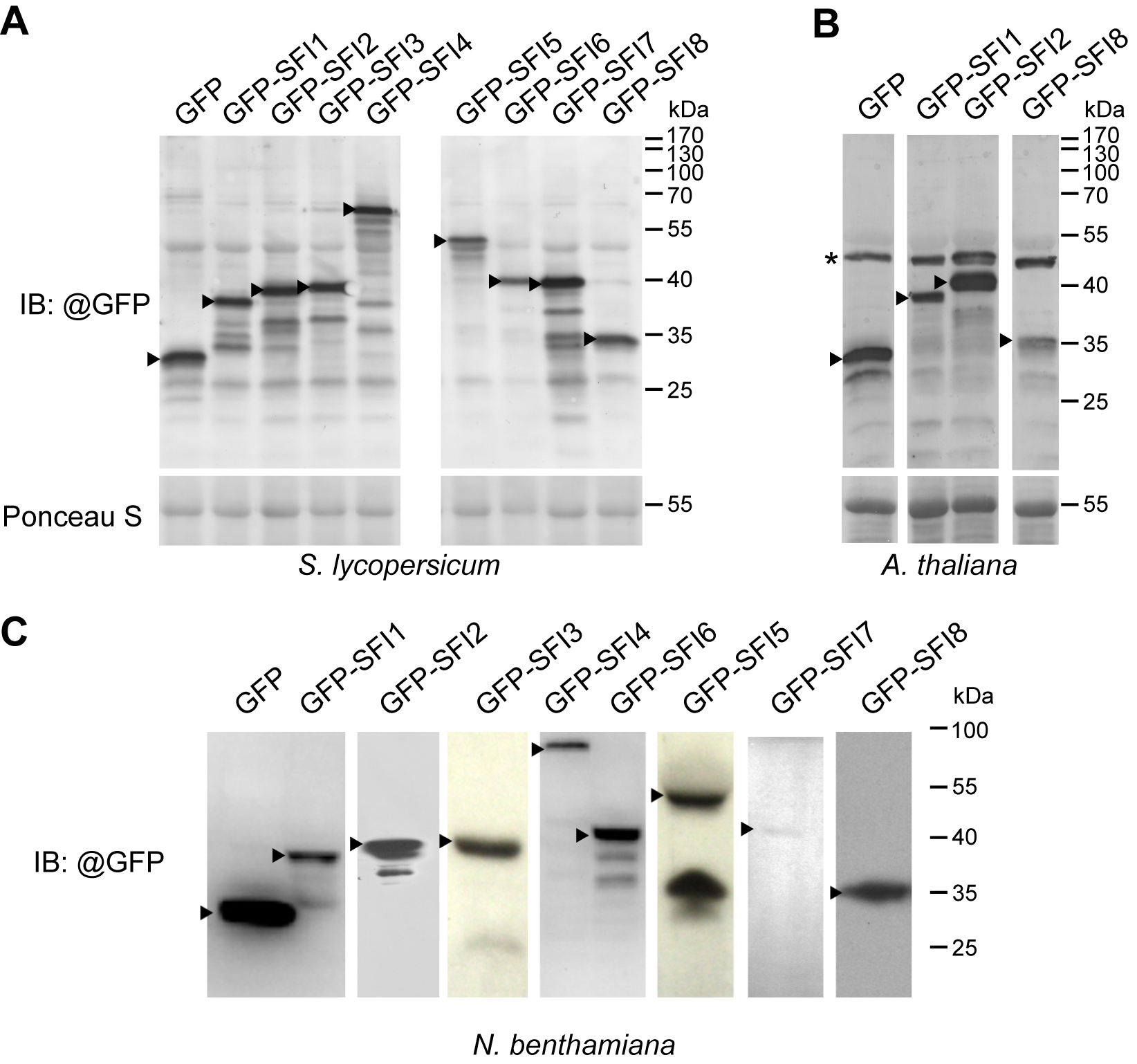

Supplement: Figure S6 — Expression profile of N-terminally GFP-tagged SFI effectors in protoplasts (A, B) and N. benthamiana leaves (C). Immunoblotting with anti-GFP antibody was carried out on protoplast samples from S. lycopersicum (A) and A. thaliana (B) 24 h post-transfection and on N. benthamiana (C) leaf extracts 48 h post-inoculation with A. tumefaciens. Signals corresponding to the different GFP fusion proteins are pointed out with an arrow. The asterisk indicates a non-specific signal. All effectors have the expected apparent molecular weight. Partial protein degradation was observed in some samples. The experiment is representative of two to three repeats. Ponceau S staining served as a loading control. (TIF) [file ppat.1004057.s006.tif]

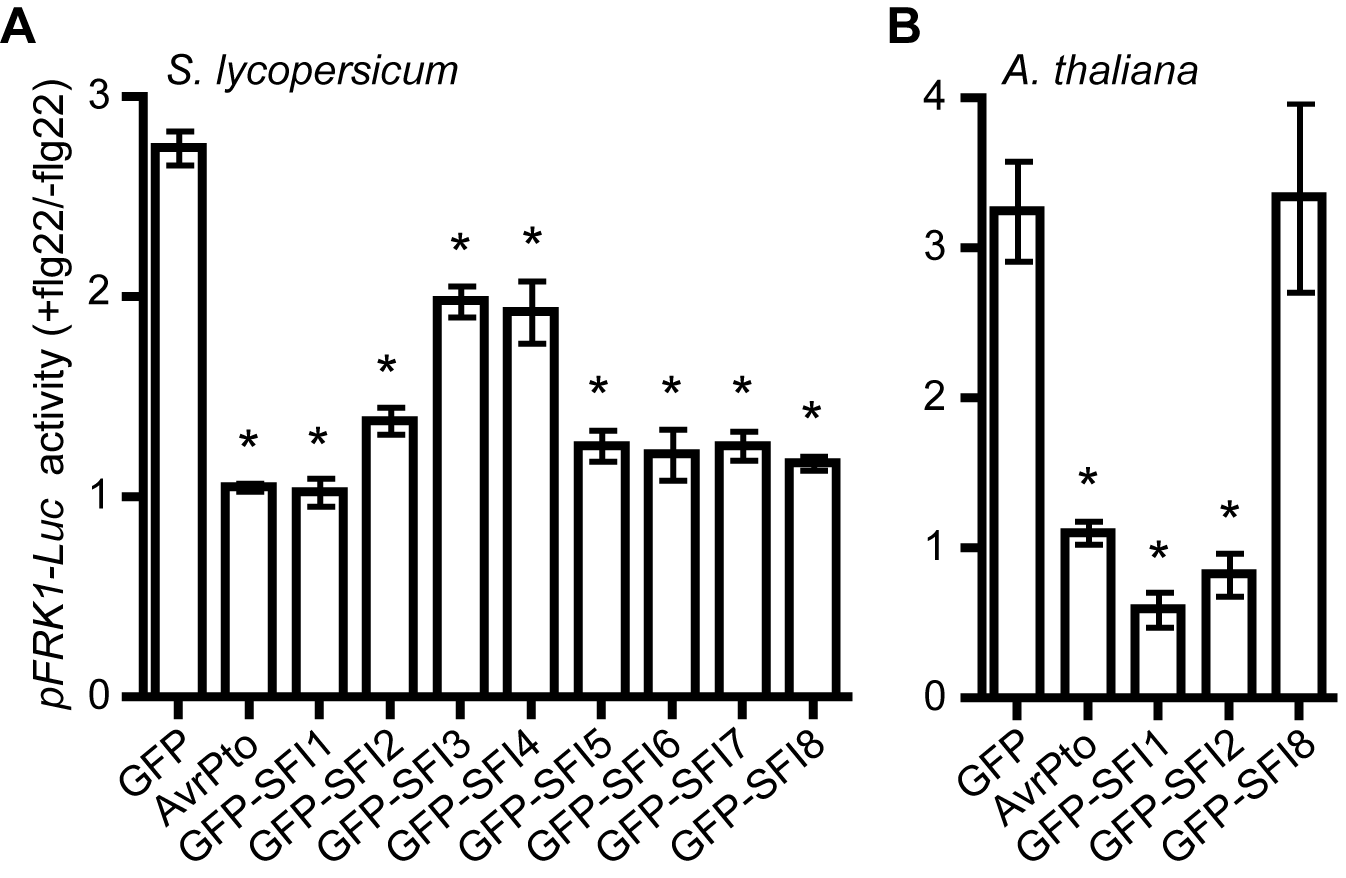

Supplement: Figure S7 — Luciferase reporter gene assay in protoplasts expressing N-terminally GFP-tagged SFI effectors. (A, B) Mesophyll protoplasts from S. lycopersicum (A) and A. thaliana (B) were used and experiments and statistical analysis were carried out as described in Figure S1. Mean values ± SEM are from at least three independent experiments. (TIF) [file ppat.1004057.s007.tif]

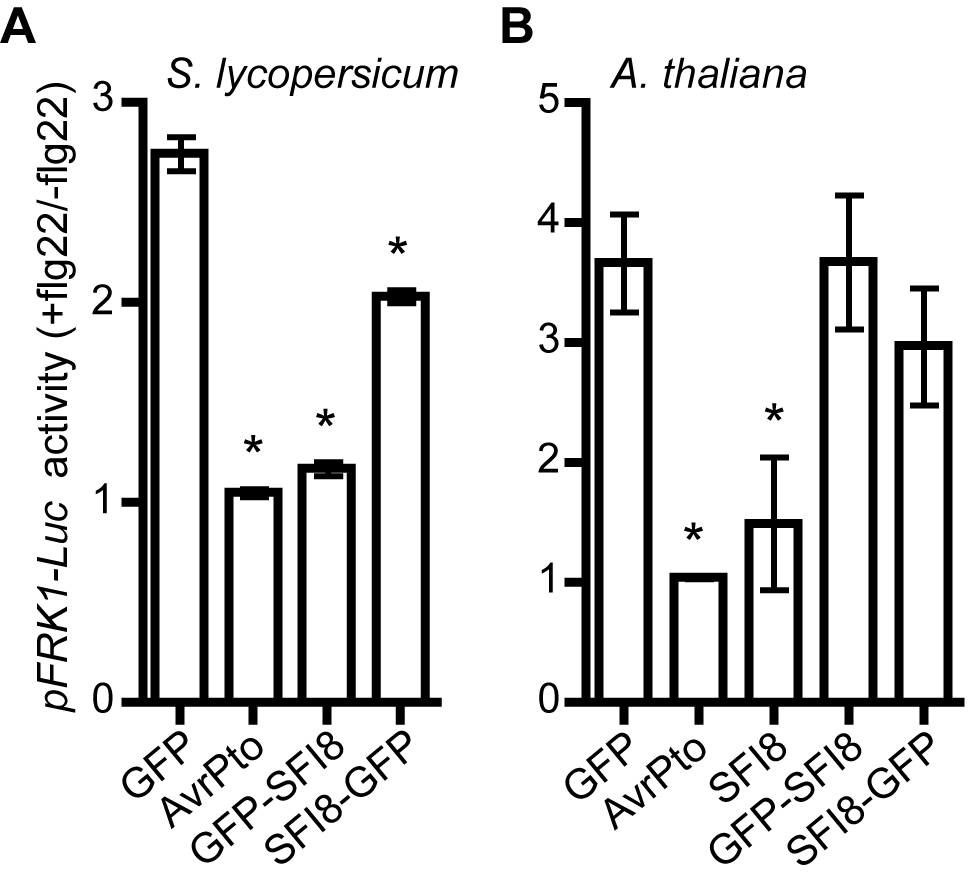

Supplement: Figure S8 — Luciferase reporter gene assay in protoplasts expressing the N- and C-terminally GFP-tagged SFI8/AVRblb2 (A, B). Mesophyll protoplasts from (A) S. lycopersicum and (B) A. thaliana were used and experiments and statistical analysis were carried out as described in Figure S1. Mean values ± SEM are from at least three independent experiments. (TIF) [file ppat.1004057.s008.tif]

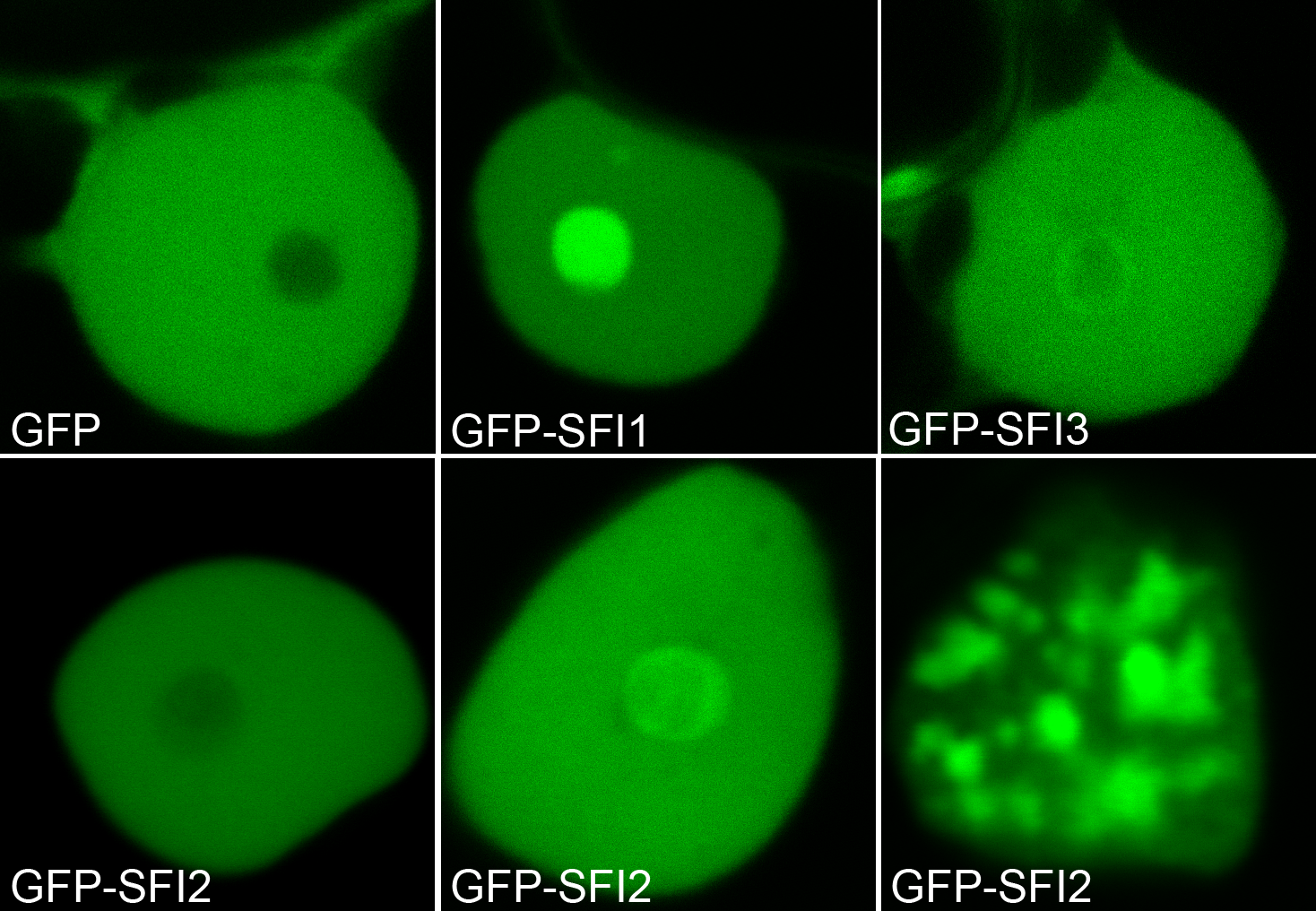

Supplement: Figure S9 — Sub-nuclear localization in N. benthamiana of SFI effectors. Typical confocal microscope close-up images of nuclei in N. benthamiana leaf cells expressing free GFP (GFP) as a control and N-terminally GFP-tagged SFI effectors (SFI numbers indicated). (TIF) [file ppat.1004057.s009.tif]

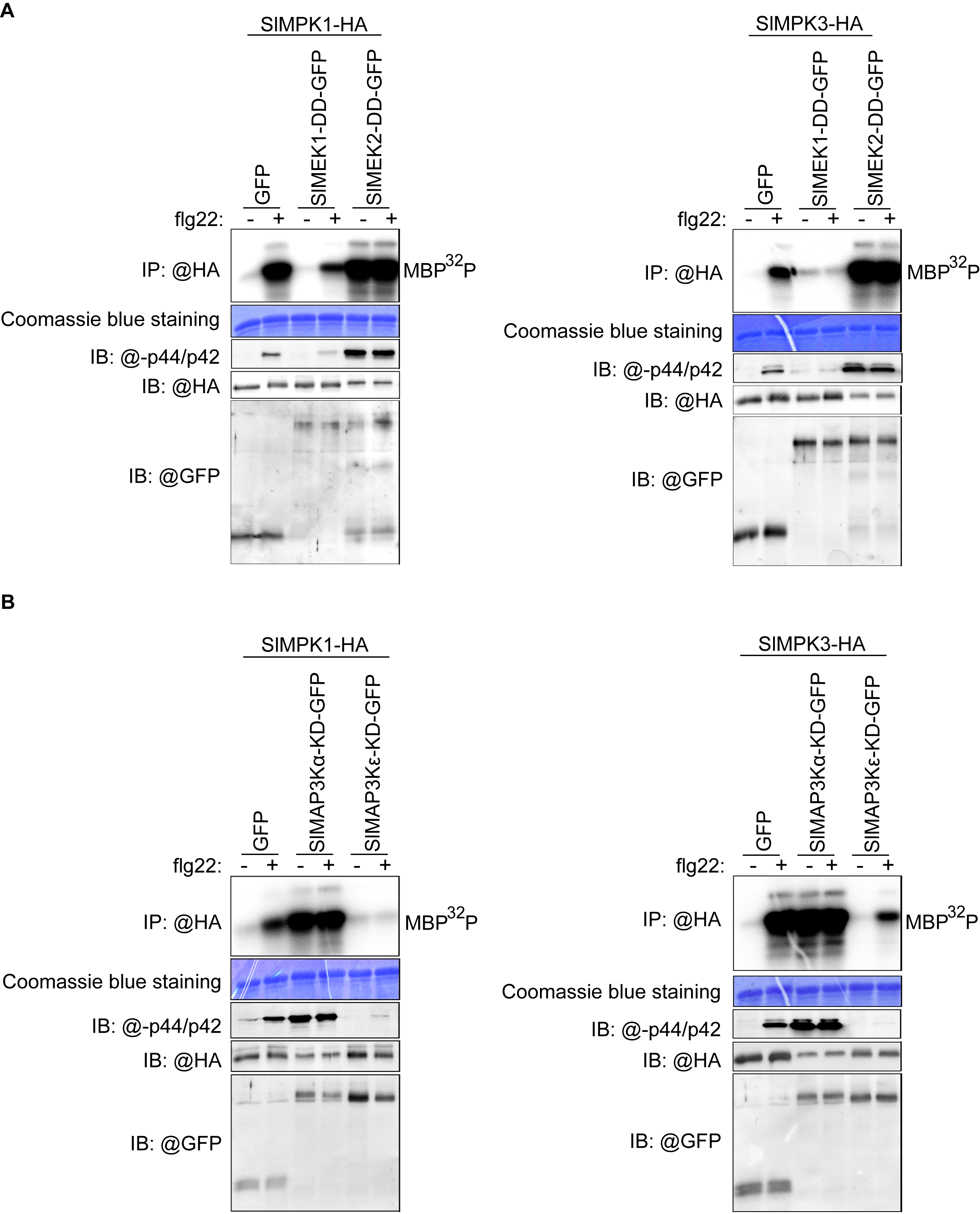

Supplement: Figure S10 — MAP kinase in vitro kinase assay in S. lycopersicum protoplasts. (A) GFP or constitutively active MAPK kinase with C-terminal GFP tag (SlMEK1-DD-GFP and SlMEK2-DD-GFP) were co-expressed with hemagglutinin (HA)-tagged S. lycopersicum MAP kinase SlMPK1 or SlMPK3. (B) GFP or the active kinase domain of MAPKK kinase with C-terminal GFP tag (SlMAP3Kα-KD-GFP and SlMAP3Kε-KD-GFP) were co-expressed with hemagglutinin (HA)-tagged S. lycopersicum MAP kinase SlMPK1 or SlMPK3. (A, B) HA-tagged MAP kinase were immunoprecipitated with anti-HA antibody for an in vitro kinase assay with [γ-32P] ATP and myelin basic protein as phosphorylation substrate (MBP32P - upper panels). Endogenous MAP kinase activation was detected with antibody raised against activated MAP kinase p44/p42 (middle panels). The lower panels present immunoblots with anti-HA and anti-GFP antibodies showing the expression of HA- and GFP-tagged proteins, respectively. Coomassie blue staining served as a loading control. The experiments are representative of at least two repeats. (TIF) [file ppat.1004057.s010.tif]
